# Clobetasol and Halcinonide Act as Smoothened Agonists to Promote Myelin Gene Expression and RxRγ Receptor Activation

**DOI:** 10.1371/journal.pone.0144550

**Published:** 2015-12-10

**Authors:** Giampiero Porcu, Eliseo Serone, Velia De Nardis, Daniele Di Giandomenico, Giuseppe Lucisano, Marco Scardapane, Anna Poma, Antonella Ragnini-Wilson

**Affiliations:** 1 Department of Biology, University of Rome "Tor Vergata", Rome, Italy; 2 Department of Translational Pharmacology, Fondazione Mario Negri Sud, S. Maria Imbaro (CH), Italy; 3 Department of Life, Health and Environmental Sciences, University of L'Aquila, L’Aquila, Italy; 4 Center for Outcomes Research and Clinical Epidemiology, Pescara, Italy; 5 Dipartimento di Scienze Mediche di Base, Neuroscienze ed Organi di Senso, Università di Bari Aldo Moro, Bari, Italy; Instituto Cajal-CSIC, SPAIN

## Abstract

One of the causes of permanent disability in chronic multiple sclerosis patients is the inability of oligodendrocyte progenitor cells (OPCs) to terminate their maturation program at lesions. To identify key regulators of myelin gene expression acting at the last stages of OPC maturation we developed a drug repositioning strategy based on the mouse immortalized oligodendrocyte (OL) cell line *Oli-neu* brought to the premyelination stage by stably expressing a key factor regulating the last stages of OL maturation. The Prestwick Chemical Library^®^ of 1,200 FDA-approved compound(s) was repositioned at three dosages based on the induction of Myelin Basic Protein (MBP) expression. Drug hits were further validated using dosage-dependent reproducibility tests and biochemical assays. The glucocorticoid class of compounds was the most highly represented and we found that they can be divided in three groups according to their efficacy on MBP up-regulation. Since target identification is crucial before bringing compounds to the clinic, we searched for common targets of the primary screen hits based on their known chemical-target interactomes, and the pathways predicted by top ranking compounds were validated using specific inhibitors. Two of the top ranking compounds, Halcinonide and Clobetasol, act as Smoothened (*Smo*) agonists to up-regulate myelin gene expression in the *Oli-neuM* cell line. Further, *RxRγ* activation is required for MBP expression upon Halcinonide and Clobetasol treatment. These data indicate Clobetasol and Halcinonide as potential promyelinating drugs and also provide a mechanistic understanding of their mode of action in the pathway leading to myelination in OPCs. Furthermore, our classification of glucocorticoids with respect to MBP expression provides important novel insights into their effects in the CNS and a rational criteria for their choice in combinatorial therapies in de-myelinating diseases.

## Introduction

Multiple sclerosis (MS) is an autoimmune demyelinating dysfunction causing inflammation, oligodendrocyte cell death and, consequently, axonal degeneration. Remyelination of axons occurs spontaneously at early stages of MS disease progression, while when disease progresses OLs arrest in a pre-myelination state. The reasons for inadequate CNS remyelination at chronic MS lesions is largely unknown [1–3]. A number of immunomodulators that effectively control the relapse number and intensity has been introduced in the clinic, but their function on remyelination processes remains to be clarified. To improve the regenerative properties of current MS treatments it is important to clarify how Oligodendrocyte Precursor Cell (OPC) differentiation and maturation occur at demyelinated lesions in the adult brain and to identify novel compounds acting in this process [4–7]. The identification of compounds acting on myelin gene expression in pre-myelinating oligodendrocytes will help in clarifying also the mechanism of how myelination occurs in adult CNS and during aging, since remyelination defects, such as those in chronic MS patients, resemble those occurring during human aging [8, 9].

The major source of myelin in the adult brain comes from developmentally committed oligodendrocytes (OLs). They originate from OPCs that proliferate and migrate into the region of lesions before differentiating into post-mitotic premyelination OLs [4, 6]. OPC proliferation and differentiation occur throughout life in normal individuals and increase upon demyelination or injury [1, 4]. Axon-glial communication, by providing trophic support to neurons, remains critical for long-term axonal integrity during the entire life span [10, 11]. The process of OPC-mediated myelination has been extensively studied during embryogenesis, but accumulating evidence suggests that OPC differentiation and OL maturation programs during remyelination do not necessarily recapitulate those occurring during embryogenesis [4, 6, 12–16]. Thus, in addition to identifying novel compounds in the appropriate OL cellular model, it is also important to clarify the molecular machinery regulating OL maturation during remyelination.

Insufficient remyelination in chronic Relapsing Remitting MS (RRMS) patients is linked to the partial or absence ability of post-mitotic OPCs, that have migrated and differentiated to complete their maturation process and express myelin genes at lesions [4, 11, 12, 16, 17]. The rapid mobilization of OPCs to the demyelinated area following injury is temporally and spatially orchestrated by a complex panel of lineage-specific transcriptional activators that promote OPC migration and differentiation at lesions [18]. The Myelin Regulatory Factor (MRF) is a critical transcriptional regulator required for CNS myelination. MRF expression is essential for OL maturation being required for the expression of the vast majority of the CNS myelin genes, but not for OPC specification or differentiation *per se* [19]. These factors compete with signals that inhibit OPC differentiation such as the canonical P-catenin-Wnt Notch1-Jagged1 signaling pathway and LINGO-1 in a manner that is still not well understood [6, 20]. Global expression profile studies aimed at identifying positive regulators of remyelination show that Retinoic x Receptor gamma (RxRγ) up-regulation is associated with OPC differentiation and remyelination at demyelinated lesions [7, 21].

The large-scale search for drugs able to promote the final stage of OL maturation has been limited thus far by the lack of an appropriate cellular model. Linage-specific OPCs differentiated up to the premyelination stage would be the cells of choice for remyelination studies. However, linage-specific primary OPC culture isolation and propagation technologies are complex and their use for large screening studies is limited by the difficulties in standardization of their culturing and maintenance conditions [17, 22]. Moreover, the cellular origin of OPCs recruited at lesions during remyelination is still under debate [7–9]. Primary OPC cultures, of rat or mouse origin, are typically heterogeneous in their composition and show different maturation stages, slow metabolism, and pro-apoptotic behavior [23–27]. Immortalized oligodendrocyte cell lines of mouse and human origin, *Oli-neu* [27] and *HOG* [25], respectively, were previously created to overcome OPC culturing difficulties. They were used extensively to clarify mechanistic aspects of myelination up to the stage of axon wrapping [23–27]. The *Oli-neu* cell line can be differentiated using chemical inducers but their use in phenotypical screens to identify compounds acting in the last stages of maturation has been limited thus far to a search for compounds able to promote their differentiation [22]. Here we exploited the key role of MRF in promoting final stages of OL maturation [19, 28] to bring the *Oli-neu* cell line to the appropriate stage of OL commitment to express myelin genes upon drug treatment. MRF expression is essential to unlock many, albeit not all, of the appropriate OL lineage-specific set of transcription factors required to promote the last steps of OL maturation. Specifically, MRF is expressed after Sox10, Olig1 and Olig2 induction and regulates only a subset of genes normally induced during OL differentiation [19, 28, 29]. In our screening set-up, MRF was stably expressed in the *Oli-neu* cell line (hereafter-named *Oli-neuM)* to promote their differentiation up to the premyelination stage without the addition of chemical compounds. *Oli-neuM* was used to screen the Prestwick Chemical Library^®^ of over 1,200 Food and Drug Administration (FDA)-approved small molecules *(* www.PretswickChemicals.com) for their ability to up-regulate MBP expression using the automated microscopy Scan^R^ platform (Olympus). The chemical-protein interactome of the hits was established after the primary screen to identify common drug targets. This allowed us to delineate the pathway(s) affected by hit drugs. The hypotheses were than validated in reproducibility tests and further validated for the top ranking drugs using biochemical and quantitative PCR methods.

Twenty three compounds belonging to three main classes were identified. We show that the GCs present in the PrestwickChemicalsLibrary^®^ of FDA-approved drugs can be divided into three groups according to their activity on myelin gene expression. Fluorinated glucocorticoids (FGCs) were the most highly represented class among the top hits, among them Clobetasol and Halcinonide. Target identification was performed using data mining bioinformatics tools and validated using biochemical assays for two of the top ranking compounds, Clobetasol and Halcinonide. We show that they induce MBP expression by acting via *Smoothened* (Smo) and by up-regulating RxRγ nuclear receptor expression via a pathway that has yet to be defined. The importance of these findings for the clinical use of group I GCs in demyelinating disease is discussed.

## Materials and Methods

### Materials, plasmid construction and cell lines

Compounds used for primary and secondary screening were obtained from the Prestwick Chemical Library^®^ (www.PretswickChemicals.com). The MyrfpCMV6 plasmid, expressing the mouse MRF (NM_001033481) cDNA http://www.origene.com/mouse_dna/NM_001033481/MC223625/Gm98.aspx) was obtained from ORIGENE. The Src and RIP2 kinase inhibitor PP2 (ab120308) was from ABCAM. The retinoic x receptor inhibitor UVI3003 (TOCRIS Bioscience) was a gift from Dr. Enrico Garattini (Consorzio Mario Negri, Milano Italy). Cyclopamine (Sc 200929) and Itroconazole (Sc 205724) were purchased from Santa Cruz and dexamethasone from Sigma (D4902-100MG). The isolation of the *Oli-neu* cell line was described previously [27] and was a gift from Dr. Lopez- Guerrero, following authorization by Dr. J. Trotter. The characteristics of the *Oli-neu* cell line are described in [25]. Typically, *Oli-neu* cells were maintained in growth medium (GM) composed of DMEM (Gibco) supplemented with 10% fetal calf serum (FCS, Euroclone), 2 mM L-glutamine (Gibco), PENSTREP 1X (15140–122; Gibco^™^), 0.1% sodium pyruvate (Gibco), and 15 mM Hepes (Sigma) at 37°C in 5% CO_2_. Differentiation medium (DM) was DMEM supplemented with 2 mM L-glutamine PENSTREP 1X (15140–122 Gibco^™^) 0.1% sodium pyruvate (11360–070 Gibco), N2 supplement 1X (175020–01 Gibco^™^), 60 nM Triiodothyronine (T3, Sigma), 53.7 ng/ml progesterone (Sigma). ***Oli-neuM* isolation**: *Oli-neuM* is a clone of the *Oli-neu* cell line transfected with the plasmid MyrfpCMV6 (ORIGINE), obtained as indicated below: 5x10^5^ *Oli-neu* cells were plated in a 25 cm^2^ flask containing GM media and left to adhere for 48h prior to being transfected with 12.5 μg of the MyrfpCMV6 plasmid using Lipofectamine 2000 (Invitrogen) according to the manufacturer's instructions. After 48h incubation in a humidified chamber at 37°C in 5% CO_2_, the transfection media was washed out and fresh GM media with 500 μg/ml G418 was added. Cells were incubated in this selective medium for 72h prior to being harvested and washed, and the cell pellet was resuspended in fresh GM media at a concentration of 10^6^ cell/ml. Then 1.5x10^6^ cells were replated in a 75 cm^2^ plate in 20 ml GM containing 500μg/ml G418 and the cells were further incubated in a humidified chamber at 37°C in 5% CO_2_ for 48h. Samples were frozen for long-term storage. Typically, after thawing, the *Oli-neuM* cell line was passed 8 to 10 times in GM media supplemented with 500 μg/ml G418 prior to performing experiments. To verify MRF expression in *Oli-neuM* clones RT-PCR reactions were performed as previously described [30] using the GAPDH gene as an endogenous control, and the following primers MRF Fw:CAACCCCGTCACGGTCAAA; MRF Rv: GCCCTTCTTGCGCATGTT; GAPDH Fw:TGTGTCCGTCGTGGATCTGA; GAPDH Rv: CCTGCTTCACCACCTTCTTGA. Primers were designed using the Primer Express^®^ software from Applied Biosystems (Foster City, CA). The amplification efficiency of each pair of primers was tested using 5 serial dilutions of cDNA derived from *Oli-neuM* RNA as template and amplification under standard conditions (see below). Amplification reactions were performed in a volume of 10 μl, using 300 nM of each primer, 3 μl cDNA and SYBR Green PCR Master Mix (Applied Biosystems, Foster City, CA). Alternatively 4 μg of DNase-treated RNA was used for reverse transcription, performed using random primers and Taqman^®^ Reverse Transcription Reagents (Applied Biosystems, Foster City, CA) according to the manufacturer's instructions. The RT-PCR amplification conditions were 50°C for 2 min, 95°C for 10 min, followed by 40 cycles of [95°C for 15 sec and 60°C for 1 min]. Each amplification was run in triplicate and replicated at least twice using independently isolated RNA. Relative expression levels were calculated automatically by the ABI Prism^®^ 7900 HT software (Applied Biosystems, Foster City, CA) with the comparative ΔΔCt method using the GAPDH gene as the endogenous control (see statistical analyses below for further details).

### Compound library screening: criteria and general methods

Cell culturing, plating, drug treatment and immunofluorescence methodologies were previously described [30, 31]. Preliminary tests were made to determine the optimal conditions for drug screening, namely: 1) optimal growth conditions prior to drug exposure; 2) drug doses to score endogenous MBP levels in the *Oli-neuM* cell line and time of exposure; 3) the reproducibility and statistical significance of treatment; 4) threshold settings to detect MBP protein level changes by using the Scan^R^ (Olympus) automated microscopy platform for acquisition and data analyses. The Prestwick Chemical Library^®^ of FDA-approved drugs was used (http://www.prestwickchemical.com/prestwick-chemical-library.html). Library compounds were purchased at 10 mM and pre-diluted in 0.5% DMSO to a concentration of 0.2, 2 or 5 mM (library stock solutions). Compounds were finally added to screening plates containing DM at 1, 10, or 25 μM final concentration in 0.5% DMSO, using automated pipets. The dosage-dependent response was evaluated in each of the 62 plates used for the screen. Dexamethasone (D4902; SIGMA) was used as positive control in each plate and was added at a concentration of 1, 10 or 25 μM in 0.5% DMSO. In screening procedure, typically, *Oli-neuM* cells were plated in 96-well Greiner-Bio-One plates, pre-coated with Fibronectin from human plasma (Sigma) as previously described [30], in growth medium (GM) for 48h. GM was removed prior to addition of DM supplemented with 0.5% DMSO (negative control) or of the indicated drugs. Cells were further incubated for 48h prior to being fixed, fluorescently stained with Hoechst (to detect nuclei) as previously described [30]. Positive and negative controls were distributed and swapped on the screening plates to eliminate local signal drifting. Acquisition and data quantification were performed using the ScanR microscopy platform (Olympus) equipped with a 20X UPLSAPO NA = 0.75 objective (Olympus) as previously described [30, 31]. Analyses: MAIN object was set based on the Hoechst nuclear signal visualized with DAPI Filter. Anti-MBP (α-MBP; MCA409S; Serotec) and Alexa488-conjugated secondary antibody (A11006 Invitrogen) were used to detect MBP and the mean FITC values (± CV) detected on MAIN/well were used for MBP level quantification. Cell cycle distribution based on quantification of total intensity signal distribution in MAIN plotted on x and y axes. Cellular morphological parameters (area, perimeter and elongation), were also acquired routinely for each sample to determine drug effects on cell morphology and cell cycle. Arborisation parameters based on morphological parameter based on the mean signal intensity distribution of actin in the cytosol, visualized using anti-actin antibody (A2066; Sigma) and Alexa546-conjugated secondary antibody (A11035 Invitrogen).

### Total RNA extraction and RT-PCR

*Oli-neuM* cells were plated in 6-well plates in GM supplemented with G418 (500 μg/ml) for 48h. After removal of the media the cells were incubated with 2.5 ml of DM and the indicated GC compound at a final concentration of 10 μM for the time indicated in the text. Total RNA was extracted using the RNeasy Mini Kit (Qiagen) according to the manufacturer’s instructions. RT-PCR was performed using the fluorescent TaqMan methodology and the ABI PRISM Fast 7500 Sequence Detection System following the manufacturer’s instructions (Applied Biosystems, Foster City, CA). Ready to use, predesigned primer and probe sets (Applied Biosystems) for mouse MBP (Mm01266402_m1), PLP1 (Mm00456892_m1), CNP (Mm01306640_m1), MOG (Mm00447824_m1), RxRγ (Mm00436411_m1), Gli1 (Mm00494654_m1), NR3C1 (Mm00433832_m1) and the housekeeping gene GAPDH (Mm99999915_g1) were used according to the manufacturer's guidelines. GAPDH was considered as a reference gene for normalization and relative quantification values were calculated using the 2^-ΔΔCt^ method. The comparative Ct method of relative quantification was performed to determine the fold change in expression. This was done by first normalizing the resulting threshold cycle (Ct) values of the target mRNA to the Ct values of the reference control. The ΔCt of target mRNA was further normalized with the calibrator (untreated control). The fold change in expression was than obtained with the equation 2^-ΔΔCt^ and data were plotted on the graph by using GraphPad Prism (GraphPad Software, Inc.). When SYBR Green-based RT-PCR was used Oli-neuM cells were treated with 10 μM of each indicated GC and 1 μM UVI 3003 was added, where indicated, and 48h treatment was performed. Total RNA was extracted using Trizol Reagent (Invitrogen) and reverse transcribed into cDNA according to the manufacturer’s instructions. Real-Time PCR was performed in triplicate using SYBR Green PCR Master Mix (Applied Biosystems) in a Fast 7500 Sequence Detection System (Applied Biosystems, Foster City, CA) as previously described. Primer pairs used for the reactions used in SYBR Green RT-PCR analysis: MBP fw TACCCTGGCTAAAGCAGAGC; MBP rv GAGGTGGTGTTCGAGGTGTC; RxR fw AGGCAGGTTTGCCAAGCTTCTG; RxR rv GGAGTGTCTCCAATGAGCTTGA; GAPDH fw TGTGTCCGTCGTGGATCTGA; GAPDH rv CCTGCTTCACCACCTTCTTGA. The Cycle Threshold (Ct) values of the target genes were normalized for GAPDH and the relative mRNA expression is reported as fold induction over the baseline as (Applied Biosystems, Foster City, CA). The dissociation curves of primer pairs used showed a single peak. Three samples per reaction and two biological replicates were tested and fold changes was calculated as indicated above using the comparative ΔΔCt method using the GAPDH gene as the endogenous control.

### Crude extract preparation and immunoblot analysis

Cell extracts (CE) were obtained from *Oli-neu* or *Oli-neuM* cells plated in a 6-well plate in DMEM growth medium and incubated for two days prior to being treated with DM alone or addition of the drug library vehicle (0.5% DMSO; mock control) or the indicated drugs as described in the text. Protein quantification and immunoblot analyses were performed as previously described [30,31] with the modifications described in Supporting Information.

### Statistical Methods

Screening data control was normalized to 100 while changes in MBP levels following drug stimulation were analyzed as relative differences from normalized controls as indicated in the text and figure legend. Mean screening values were computed as weighted means of the first two screenings, using cell number as weights. For each compound a two-tailed Student's *t* test was performed to assess differences between treatments vs. control samples. We used one-way ANOVA to compare more than one treatment at once to the same control. A P value < 0.05 were considered to be statistically significant. Post-hoc tests were performed using FDR (False Discovery Rate) correction. All analyses were performed using SAS software (Release 9.3. Cary, NC, USA). For RT-PCR data, each gene was statistically compared to GAPDH as an endogenous control, and mean values were computed for each drug treatment. ΔCt values were computed as mean absolute differences between each compound and ΔΔCt values were computed as absolute differences between sample ΔCt and control ΔCt. Unpaired two-sided t-tests for each treatment were performed to assess whether ΔCt and ΔΔCt were statistically different from 0 (Applied Biosystems, 1997. User Bulletin No. 2 ABI Prism 7700 Sequence Detection System. Applied Biosystems. Available from http://www3.appliedbiosystems.com/cms/sroups/mcbsupvort/documents/seneraldocuments/cms040980.pdf).

## Results

### Development of a cell-based assay suitable for screening library compounds stimulating MBP expression in immortalized oligodendrocyte precursor cell lines

**Fig 1:**
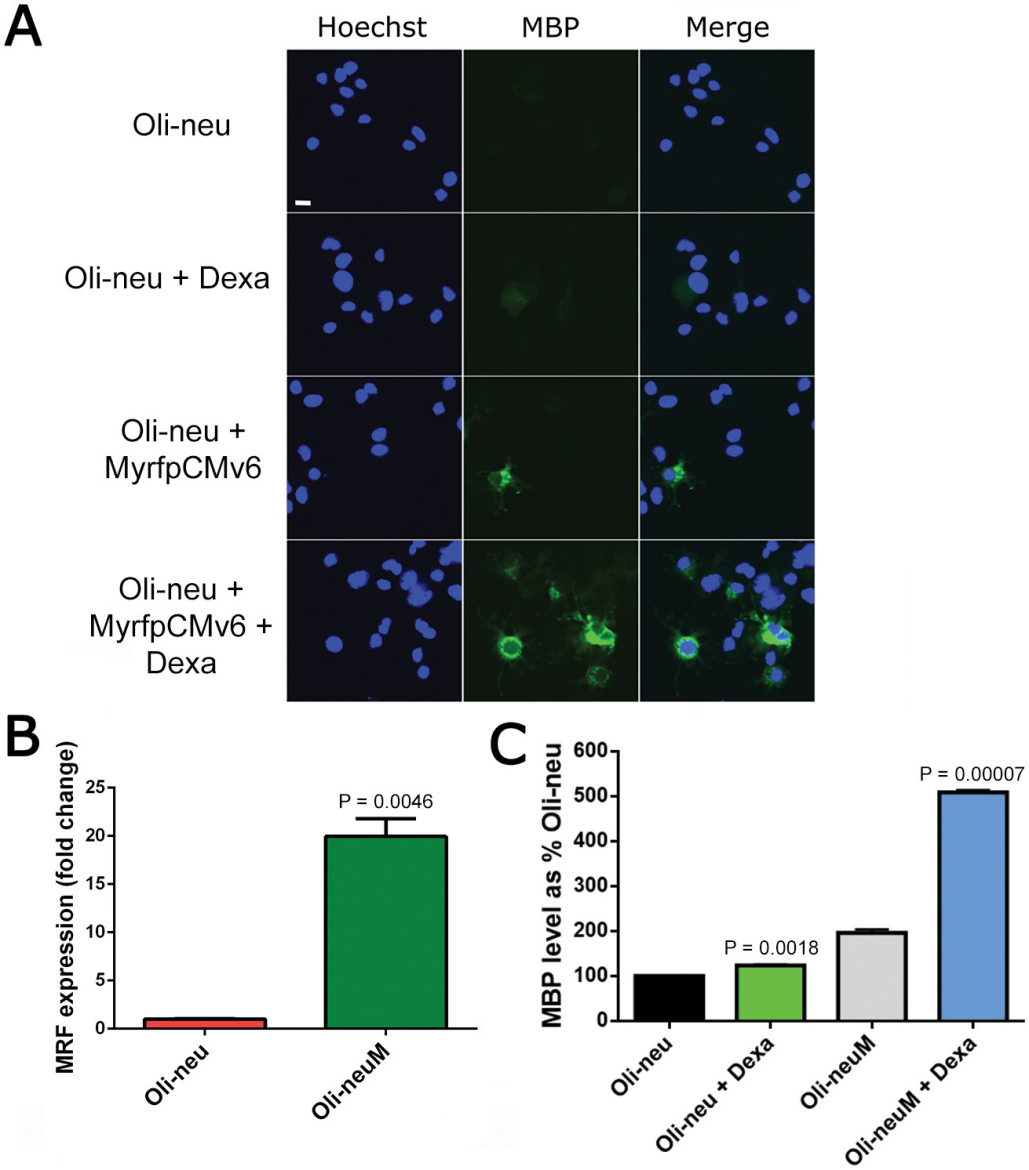
*Oli-neuM* cells express higher levels of MBP than *Oli-neu* cells. (A) *Oli-neu* cells were plated in a 96-well plate, left to adhere for two days, treated with differentiation medium (DM) prior to treatment with 10μM Dexamethasone and/or transfected with the MRF-expressing construct (MyrfpCMV6) as indicated. After two days of growth cells were fixed and stained with an α-MBP antibody (panel MBP). Nuclei were stained with Hoechst (panel Hoechst). Scale bar: 10 μm. (B) Expression of MRF in *Oli-neu* cells and in *Oli-neuM* (*Oli-neu* cells stably expressing MRF) was measured by RT-PCR. The expression level in *Oli-neu* cells was arbitrarily set at 1. Data were acquired in triplicate and are presented as the mean ± SD. Statistical significance was analysed by a two-tailed Student's *t* test. (C) MBP protein levels were measured using the Scan^R^ platform for acquisition and data analysis. *Oli-neu* or *Oli-neuM* cell lines were plated and treated as indicated in the text. Mean values ± SD of two independent experiments acquired in triplicate from the indicated cell line are plotted in the graph. The MBP protein level of the *Oli-neu* cell line was set arbitrarily at 100%. Statistical significance between dexamethasone-treated and untreated samples was analysed by a two-tailed Student's *t* test.

An immortalized mouse oligodendrocyte cell line called *Oli-neu* has been selected previously to study the mechanism of myelin gene expression and transport to membranes in OPCs [27]. Treatment with di-butyryl-cyclic (Dbc)-AMP leads to myelin gene expression and several studies showed the ability of this cell line to appropriately recapitulate the differentiation stages required for myelination allowing a mechanistic understanding of this process [22, 24–26]. Importantly, upon transplantation into demyelinated lesions, *Oli-neu* specifically ensheaths axons although a compacted myelin sheath is not formed [24, 25]. As previously mentioned, the *Oli-neu* cell line has limitations in drug screening studies aiming at the identification of compounds acting in remyelination, since such studies require bringing OPC differentiation to the premyelination stage, which requires linage-specific transcription factors. We reasoned that expression of MRF in the *Oli-neu* cell line would allow this cell line to reach the pre-myelination commitment stage, as shown for primary OLs [19, 28]. For this reason we transfected the *Oli-neu* cell line with the MyrfpCMV6 plasmid and then determined MBP levels in transfected compared to non-transfected cells using automated quantitative fluorescence microscopy (Fig 1A) and Real-Time PCR (RT-PCR, Fig 1B). Unless otherwise stated, in all automated fluorescence microscopy studies reported below, samples and controls were analyzed in parallel using the Scan^R^ (Olympus) microscopy and software platform after plating for 48h in 96-well plates. MBP expression was induced in these experiments using Dexamethasone, which is known to stimulate *Oli-neu* differentiation and thereby promote MBP expression [22]. Moreover, supporting its efficacy *in vivo*, appropriate treatment with Dexamethasone recovers the clinical defects of Experimental Autoimmune Encephalomyelitis (EAE), an inflammatory animal model for RRMS [32]. How Dexamethasone mechanistically exerts these effects is poorly understood. As expected, *Oli-neu* cells transiently expressing MRF show higher levels of MBP compared to non-transfected cells in the presence or absence of Dexamethasone (Fig 1A). Following these experiments an *Oli-neu* cell line subclone stably expressing MRF was established, as described in Materials and Methods, and named *Oli-neuM*. After confirming that the *Oli-neuM* cell line expresses MRF (Fig 1B) and responds to Dexamethasone by up-regulating MBP at levels higher than *Oli-neu* cells as estimated using immunofluorescence analyses of samples (Fig 1C), we used the Oli-neuM cell line for further studies.

### Screening compounds of the Prestwick Chemical Library^®^ for their activity on myelin basic protein expression

**Fig 2:**
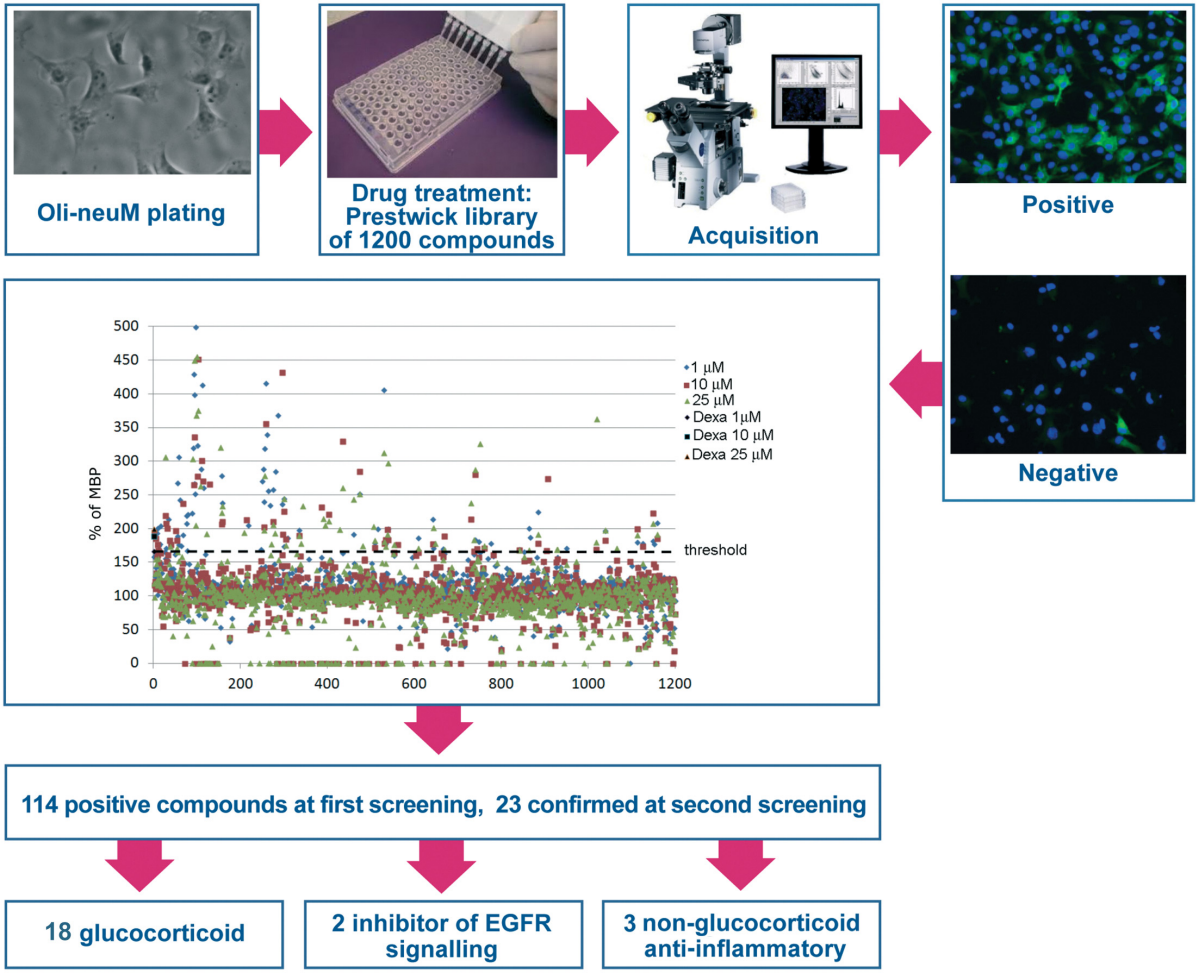
Screening flow and results. The picture illustrates the flow and the results of screening re-profiling of the over 1,200 clinically approved compounds from Prestwick Chemicals, tested on the cell line *Oli-neuM* as described in Methods. MBP protein expression was plotted on a Microsoft Excel graph for all of the compounds at the three concentrations tested together with the control Dexamethasone. In the primary screen we considered as positive only those compounds that exceeded or matched the lowest MBP protein expression observed following Dexamethasone treatment. In the secondary screen we considered as hit compounds only those drugs that were able to induce MBP protein expression better than Dexamethasone at all three concentrations tested (1, 10, 25 μM). (Dexa = Dexamethasone).

The general strategy undertaken is highlighted in Fig 2. A drug re-profiling strategy was chosen as FDA-approved small molecules libraries are composed of thousands of pharmacologically characterized biologically active compounds for which most cellular targets are known [33, 34]. We reasoned that their use, in addition to potentially providing useful drugs immediately available in the clinic, might allow the identification of networks regulating myelin gene expression in *Oli-neuM* cells, since these libraries are, in other words, enriched in compounds with agonist or inhibitory activity towards known protein targets.

Briefly, we used the Prestwick Chemical Library^®^ of 1,200 biologically active FDA-approved small molecules since this library was previously used successfully to identify pro-myelinating drugs in zebrafish [35]. The three dose-response strategy [36] was chosen for the primary drug screen in order to reduce the false positives to be analyzed in secondary and validation tests. In the primary screen we considered as hits those drugs that up-regulated MBP expression higher than Dexamethasone with at least one of the doses used. We observed that MBP induction does not occur prior to 24h after treatment, with the best time frame being between 24 and 48h. Therefore, we performed all the tests after 48h unless otherwise stated. Following preliminary tests, involving 80 randomly selected library compounds, we established plate array distribution, library compound dose range, and imaging settings, as described in Materials and Methods. Finally, each of the 1,200 library compounds was tested at 1 μM, 10 μM and 25 μM for their ability to induce MBP expression. MBP levels, DNA content and differentiation (arborisation) parameters were routinely quantified using Mean Intensity FITC, Total intensity DAPI, and morphological parameters as we previously described [30,31] and data statistically filtered for their significance as described in Materials and Methods. Typically, the chemical library and appropriate drug controls were administrated to *Oli-neuM* cells plated for 48h in 96-well plates suitable for image analysis. The image data were acquired 48h after treatment and the statistical significance of variation compared to controls (S1 Table) was evaluated as described in Materials and Methods. Following primary screening, 114 compounds were found to stimulate MBP expression at levels equal to or above that of Dexamethasone with at least one of the three concentrations used (Fig 2).

### Secondary screening confirmed 23 compounds of the primary screen in reproducibly stimulating MBP expression in *Oli-neuM* cells

**Fig 3:**
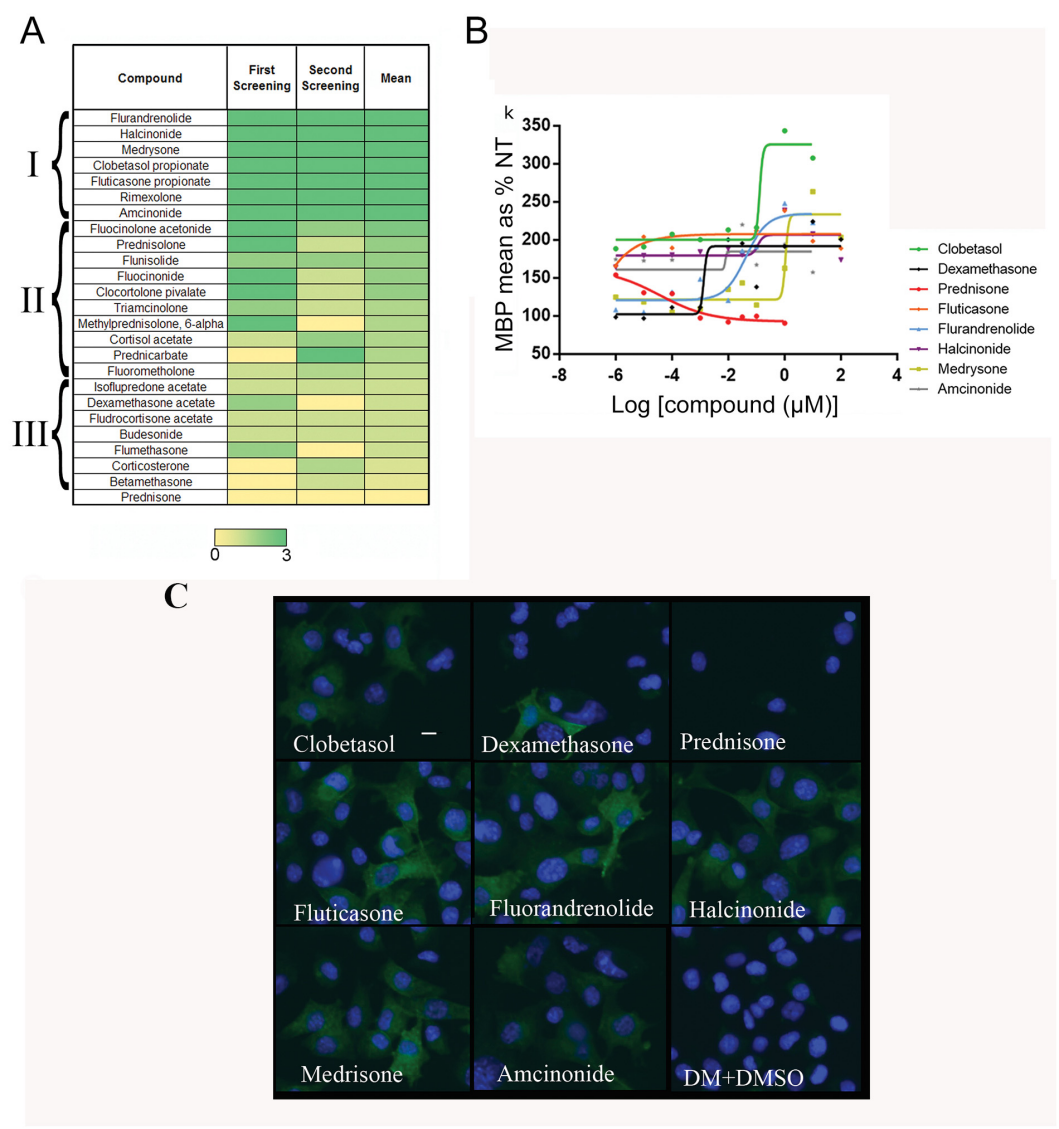
Glucocorticoids are potent myelin gene inducers. (A) Glucocorticoid classification according to their activity on MBP in both the primary and secondary screens. Intensity scale: yellow colour (0) means no increase in MBP expression over Dexamethasone; green colour means MBP induction: the stronger the induction, the more intense the green colour is represented. Higher level of MBP induction has an intensity scale 3. Based on the intensity scale, glucocorticoids were divided into three classes (black brackets). (B) Dose response curves of all positive glucocorticoids, using Dexamethasone and Prednisone as controls. Ten dose-response curves were performed over a range of concentrations up to 25 μM as indicated in the text. *Oli-neuM* MBP protein levels were calculated by Olympus Scan^R^ software analysis (more than 1,000 cells were analyzed for every condition). MBP protein levels were calculated as % increase compared to NT value. Data were acquired in triplicate (*n* = 2) and analyzed by nonlinear regression and fitted to a sigmoid dose-response using GraphPad Prism (GraphPad Software, Inc.). (C) Representative images of *Oli-neuM* untreated (DM+DMSO) or treated with 10 μM of the indicated drugs. Images were taken with a 20X objective of the Olympus Scan^R^ platform (scale bar: 10 μm).

To validate the primary screen hits we re-tested the 114 drugs at three concentrations and considered as positive only those that reproducibly act on MBP expression in a dose-dependent manner above or equal to dexamethasone. Only 23 drugs passed this reproducibility test: eighteen GCs, the ERB/GRB inhibitors Gefitinib and Erlotinib previously identified as inducers of OPC differentiation [22], and three drugs belonging to other chemical classes (Fig 2). Importantly, reproducibility tests showed that the 25 GCs present in the Prestwick Chemical Library^®^ can be divided into three major groups with respect to their ability to induce MBP expression (Fig 3A). In addition to the Fluorinated Glucocorticoids (FGCs) Clobetasol, Flurandrenolide, Halcinonide and Fluticasone, two corticosteroids used in ophthalmology (Rimexolone and Medrysone) ranked among the top GCs in dose-response and reproducibility tests (Fig 3A and 3B). Prednisone, the inactive precursor of Prednisolone [37], ranked at the bottom together with Corticosterone and Betamethasone (Fig 3A, group III). Methylprednisolone, currently used to treat a variety of neurological disorders, including MS [38], ranked in group II and Dexamethasone in group III.

To further confirm these data, we mined the literature to determine the EC50 that has been described for each hit GC based on other targets, as well as the optimal concentration for MBP activation as established by this study. We then defined a ten-dose-response curve to test GC drug activity on MBP expression in the *Oli-neuM* cell line. Clobetasol, Flurandrenolide, Halcinonide (group I) and, as expected, Dexamethasone (group III) showed a clear dose-response for MBP induction, while Prednisone was mostly inactive within this concentration range (Fig 3B, S2 Table). In top ranking GC-compounds treated cells, MBP show a cytosolic distribution and in some cases accumulation in spot like structure and membrane (Fig 3C).

**Fig 4:**
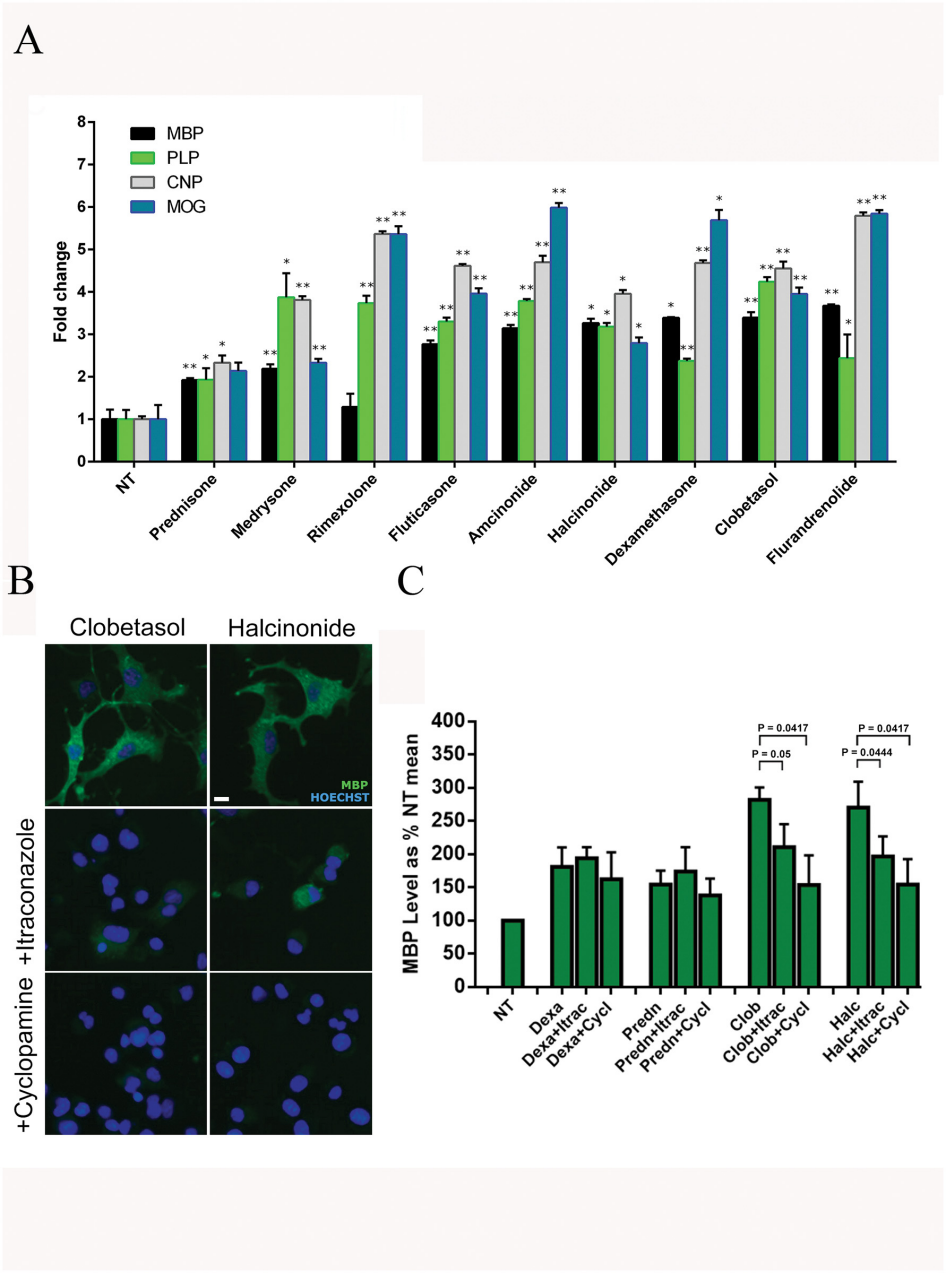
Clobetasol and Halcinonide promote Smoothened activation. (A) RT-PCR of MBP, PLP, CNP and MOG induced by 10 μM glucocorticoid treatment. All glucocorticoids significantly up-regulate all myelin genes tested with fold change induction above NT level (arbitrarily set as 1). Data acquired in triplicate are presented as the mean ± SD. Statistical significance was analyzed by a two-tailed Student's *t* test. * p<0.05, ** p≤0.01 for each gene vs. NT. MBP expression: Prednisone P < 0.0001, Medrysone, Fluticasone, Amcinonide, Clobetasol, Flurandrenolide, Dexamethasone P = 0.0016, Halcinonide P = 0.0012. PLP expression: Prednisone P = 0.0263, Medrysone P = 0.0255, Rimexolone, Dexamethasone P = 0.0001, Fluticasone, Clobetasol, Amcinonide P < 0.0001, Halcinonide P = 0.0008, Flurandrenolide P = 0.0175. CNP expression: Prednisone P = 0.0061, Medrysone, Rimexolone, Fluticasone, Amcinonide, Dexamethasone, Clobetasol, Flurandrenolide P < 0.0001, Halcinonide P = 0.0006. MOG expression: Medrysone, Rimexolone, Fluticasone, Amcinonide, Clobetasol, Flurandrenolide P < 0.0001, Dexamethasone P = 0.0006, Halcinonide P = 0.0026). (B) representative images and (C) quantification of MBP expression in *Oli-neuM* cells plated in 96-well plates containing DM for 48h and then treated with 10 μM of the indicated FGC with or without 150 nM Cyclopamine or 5 μM Itraconazole and further incubated for 48h prior to inspection. Images were taken with a 20X objective using the Olympus Scan^R^ platform. More than 1,000 cells/sample were analysed. Data are plotted as the mean ± SD of three wells per analysis (*n* = 4). Statistical analysis was performed using one-way ANOVA single compound treatment and together with Itraconazole and Cyclopamine: Dexa vs Dexa+Itrac vs Dexa+Cycl = 0.3811; Predn vs Pren+Itrac vs Pred+Cycl = 0.2496: Clob vs Clob+Itrac vs Clob+Cycl = 0.0017; Halc vs Halc+Itrac vs Halc+Cycl = 0.0044. Dexa, Dexamethasone; Itrac, Itraconazole; Cycl, Cyclopamine; Predn, Prednisone; Clob, Clobetasol; Halc, Halcinonide. Scale bar: 10 μm.

To determine whether the seven top ranking GCs of group I influence not only MBP protein levels but also CNPase, DM20/PLP and MOG myelin gene expression, we performed RT-PCR (Fig 4A) and immunoblot analyses (S1 and S2 Figs). Furthermore, to evaluate their potency compared to less active GC compounds (group III), we included Dexamethasone and the inactive Prednisone in these studies. FGCs belonging to group I resulted in a significant increase in global myelin expression compared to non-treated controls (Fig 3C, NT) and to Prednisone, as determined by RT-PCR after 48h treatment. Rimexolone, Amcinonide, Dexamethasone and Flurandrenolide were the most active in inducing CNPase and/or MOG at the transcriptional level. Medrysone, Rimexolone, Fluticasone, Halcinonide, Amcinonide and Clobetasol also activated DM20/PLP gene expression compared to the control and Prednisone. Fluticasone, Amcinonide, Halcinonide and Clobetasol were positive for induction of expression of all myelin genes tested (MBP, PLP/DM20, CNPase and MOG) compared to controls (NT and Prednisone).

In summary, 23 drugs passed the secondary screen based on dose-dependent reproducibility tests. Dexamethasone and two ErbB/GRB inhibitors, Gefitinib and Erlotinib, were previously shown to promote differentiation of *Oli-neu* cells and Rat OPCs [22, 32] and passed our secondary screen, supporting our overall set-up and screening methodology. We observed that reproducibility tests allowed to reclassify the Prestwick Chemical Library^®^ Glucocorticoids into three major groups according to their efficacy in MBP up-regulation. Of these, seven could up-regulate MBP expression at the three concentrations tested in a dose-dependent manner (Fig 3A and 3B) to levels greater than that induced by Dexamethasone (Fig 3A, group I). Thus, the class I FGCs, namely Flurandrenolide, Fluticasone, Amcinonide, Halcinonide and Clobetasol, emerged as the top-ranking pharmacological compound class in our screening set up. Given that target identification is compulsory to bring a compound till clinic, we further focused on class I FGCs group target identification and among them we choose Clobetasol and Halcinonide as they were the most representative of this group considering the score they obtained in all test performed.

### Clobetasol- and Halcinonide-mediated MBP expression requires *Smoothened* activation

Interestingly, literature mining revealed that Clobetasol, Halcinonide and Fluticasone (Fig 3A, group I) act as Smoothened (*Smo*) agonists in U20S and HEK293 cells [39]. *Smo* is a member of the seven trans-membrane family of serpentine receptors that in a canonical pathway is activated upon release from Patched *(PTCH)*, a twelve-pass trans-membrane protein that binds to Hedgehog ligands. In canonical activation pathways *Smo* acts on downstream processes by activating *Gli*-mediated gene transcription. However, non-canonical pathways have also been described [40, 41]. Although several studies have related *Smo* activation to oligodendrocyte differentiation [42–45], if and how *Smo* activation promotes myelin gene expression in pre-myelinating OPCs has never been investigated.

To determine the role of *Smo* activation in Clobetasol- and Halcinonide-mediated MBP up-regulation in *Oli-neuM* cells, we treated them with Cyclopamine and Itraconazole. Both of these compounds bind to *Smo*, but at distinct sites, inhibiting its activity [39, 46, 47]. Based on literature data [39, 46] and our own titration experiments, we used Cyclopamine at a concentration of 150 nM and Itraconazole at 5 μM. Samples were taken at 2, 4, 6, 24 and 48 hours after treatment. The mean MBP intensity observed in each sample was estimated by quantitative fluorescence microscopy analyses using the Scan^R^ acquisition and analysis platform (Fig 4B), based on the analyses of at least 16 images per well, randomly taken in treated and control samples (NT) plated in 96-well plates. Mean data of at least three wells per sample were considered for statistical analyses. Data from independent experiments (n = 4) were plotted as mean % of MBP level normalized to control (NT sample), arbitrarily set at 100% (Fig 4C). We observed that Cyclopamine significantly inhibits (P = 0.0417) MBP expression in Clobetasol- or Halcinonide-treated cells. Itraconazole was slightly less effective in the case of Clobetasol-treated cells (P = 0.05) although significantly affects Halcinonide-mediated MBP expression (P = 0.0444). We interpret the different efficacy of these two compounds as a consequence of their different sites of *Smo* binding [46, 47]. Cyclopamine or Itraconazole treatment did not significantly affect Dexamethasone-induced MBP expression, indicating that Clobetasol and Halcinonide, but not Dexamethasone, up-regulate MBP expression via a *Smo*-dependent pathway. MBP expression in Prednisone-treated cells was similarly not affected by Itraconazole or Cyclopamine treatment.

### Top ranking FGCs acting as *Smo* agonists up-regulate *RxRγ*

The next question was what is the target of the FGC-mediated signaling to the nucleus? RxRγ is a nuclear receptor whose transcription is up-regulated at demyelinated lesions and it promotes OPC differentiation and myelination [21]. Thus, it was important to establish if the selected FGCs up-regulate *RxRγ*. Moreover, the chemical-protein interaction network of our primary screen, determined using STITCH software (v.3.0, http://stitch.embl.de/), indicated that the 114 drugs that passed the primary screen targeted a limited number of proteins (listed in S3 Table), suggesting that they might act via common intermediates on myelin gene transcription. The analyses of drug target protein-protein interaction maps, using STRING software (http://string.embl.de/ version 9.1), highlighted the glucocorticoid receptor (GR) NR3C1 and JUN transcription factor as potential nuclear targets of these protein-signaling networks (S3 Fig). The GR NR3C1 has several levels of cross-talk with the Vitamin D Receptor or Pregnane x Receptor [48]. These nuclear receptors share common responsive elements in target genes and are able to form heterodimers with *RxR* [49]. Based on these data we analyzed the level of expression of NR3C1, *Gli1* and *RxRγ* in *Oli-neuM* cells using RT-PCR following treatment with the class I FGCs in comparison to untreated cells (NT), and cells treated with Dexamethasone or Prednisone.

**Fig 5:**
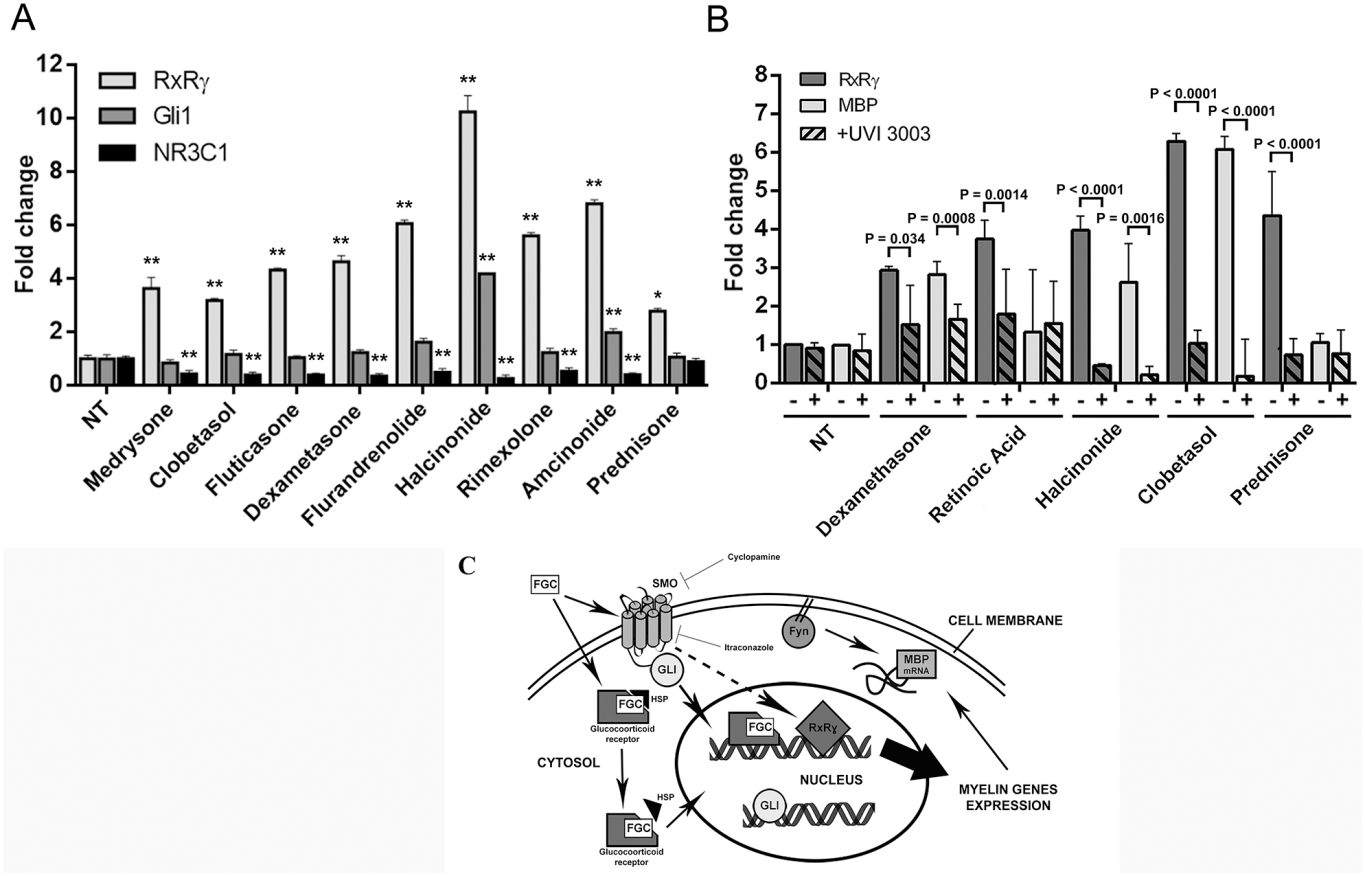
*RxRγ* expression is up-regulated by GCs. (A) RT-PCR of *RxRγ*, *Gli1* and NR3C1 expression. Data acquired were normalized using the GAPDH housekeeping gene and the NT level were arbitrarily set as 1. Data were acquired in triplicate and are presented as the mean ± SD. Statistical significance was analysed by a two-tailed Student's *t* test, comparing the fold change of each gene against its respective NT. * p<0.05, **p≤0.01 *RxRγ* expression: Prednisone P = 0.0106, Dexamethasone P = 0.0029, Clobetasol Fluticasone, Flurandrenolide, Amcinonide. P < 0.0001, Halcinonide P = 0.0007, Rimexolone P = 0.0008, Medrysone P = 0.0027. Gli1 expression: Halcinonide P < 0.0001, Amcinonide P = 0.0014. NR3C1 expression: Dexamethasone, Clobetasol, Fluticasone, Rimexolone, Halcinonide P < 0.0001, Medrysone. P = 0.0022, Flurandrenolide P = 0.0001, Amcinonide P = 0.0006. (B) RT-PCR of RxRγ and MBP expression induced by 10 μM of the indicated compounds with or without 1 μM UVI 3003 treatment. The UVI 3003 antagonist (+, where added) decreases RxRγ and MBP expression compared to *Oli-neuM* cells treated with glucocorticoid alone. Data acquired in triplicate (*n* = 2) were analyzed as described above. Statistical significance is shown for samples with (+) compared to samples without (-) UVI3003 for each treatment and were analysed by a two-tailed Student's *t* test. (C) Model of FGC mechanism of action on MBP expression in oligodendrocytes.

Surprisingly, we observed poor activation of the glucocorticoid receptor NR3C1 compared to NT and to Prednisone-treated cells, while *RxRγ* was strongly induced upon top ranking FGC treatment (Fig 5A). A significant up-regulation of *Gli1* expression was observed only upon Halcinonide and Amcinonide treatment, while all the other compounds tested had minor or no effects (Fig 5A). As an example of group I FGC mode of action, the role of RxRγ was further investigated for the case of Halcinonide- and Clobetasol-induced MBP expression. For this we performed a similar RT-PCR experiment but this time we also added UVI 3003 (3-[4-Hydroxy-3-[5,6,7,8-tetrahydro-5,5,8,8-tetramethyl-3-(pentyloxy)-2-naphthalenyl]phenyl]-2-propenoic acid; Tocris Bioscience) a RxR antagonist that displays high RxR binding affinity. We observed that UVI 3003 addition potently inhibits both Halcinonide- and Clobetasol-mediated *MBP* and *RxRγ* expression (Fig 5B). Dexamethasone-mediated MBP expression was slightly inhibited, while no significant effects were observed for Prednisone-mediated MBP activation, although also in this sample UVI 3003 inhibited *RxRγ* expression (Fig 5B).

In summary, Clobetasol and Halcinonide act in MBP expression prevalently through RxRγ activation while Dexamethasone appears to promote MBP expression via a pathway less affected by RxRγ activation. These data support the idea that FGCs of class III might operate in MBP activation via different pathways than those of class I. Interestingly, the action of Halcinonide and Clobetasol on MBP expression can be differentiated by the relative amount of *RxRγ* and *Gli1* gene expression. None of them significantly up-regulate NR3C1 compared to controls in our assay system.

## Discussion

Here we showed that GCs can be broadly classified in three groups according to their activity on MBP expression. Class I glucocorticoids, namely Clobetasol, Fluorandrenolide Halcinonide, Medrysone, Fluticasone, Rimexolone and Amcinonide, are the most potent inducers of myelin expression among the 1,200 FDA-approved drugs of the PrestiwickChemicalsLibrary^®^ in *Oli-neu* cell line stably expressing the MRF gene. The target of Clobetasol and Halcinonide at *Oli-neuM* membrane is the Sonic Hedgehog receptor *Smoothened* and their action results in *RxRγ* expression up-regulation.

A limiting factor in identifying compound(s) acting in oligodendrocyte-mediated myelination thus far has been the lack of a tractable cell-based assay in which to perform large scale screening studies [17] as well as a poor understanding of the origin of OPCs migrating at re-myelination lesions [8]. Here we have shown that by ectopically expressing MRF in the immortalized cell line *Oli-neu* it is possible to use this cell line for large phenotypical screens aiming at the identification of regulators of last stages of myelination in premyelinating OLs. While this manuscript was in submission Najm et al. [50] reported the identification of Clobetasol in a repositioning screen performed using mouse Epiblast Stem Cell (EpiSC)-derived OPCs and the NIH library collection I and II. To analyze the overlap between their and our screenings hits we performed Venn analyses using the publically available Comparative Toxicogenomics Database (MyVenn tool available at http://ctdbase.org/tools/myVenn.go?q). This analysis revealed that 12 drugs passed our secondary screen and the primary screen criteria of Najm et al. [50], namely: Amcinonide, Clobetasol, Clotrimazole, Dexamethasone, Flunisolide, Fluocinolone, Fluorometholone, Fluticasone, Hydrocortisone, Prednisolone, Triamcinolone and Azathioprine. Importantly, Najm et al. [50] reported Clobetasol validation in PLP- and MOG- EAE animal model for relapsing remitting and chronic MS and in lysolecityne re-meylination animal models. The fact that completely independent drug screens, performed with different OPC cellular models and drug libraries, produced a similar shortlist of compounds promoting myelination further confirmed that Clobetasol is a potential active drug to be used in the regenerative medicine therapy of demyelinating diseases affecting the CNS. Further characterization of *Oli-neuM* differentiation markers, compared to the original *Oli-neu* cell line will be required to consolidate the idea that *Oli-neuM* provides a cellular model for high throughput screening of compounds acting at the final stages of OL maturation prior to MBP expression. In any case, *Oli-neuM* cell line will be a valuable tool to further dissect their mode of actionings. Further confirming the quality of our assay criteria and set up, our screening strategy recapitulated also other previous findings [22, 35, 50], as we selected among the top ranking compounds the Erb/Grb inhibitors Gefitinib and Erlotinib. We did not identify *13-cis* retinoic acid or PKA activators described in Joubert et al. [22] as he used differentiation screening criteria to identify compounds acting in myelin gene expression in *Oli-neu* cell line while we set our screen on selecting compounds acting after MRF expression [19], thus is conceivable that we found a greater overlap with drug hits reported by Najm et al. [50]. Our data are fully consistent with the previously reported role of MRF in OL maturation but not OPC differentiation [19, 29].

Identification of drug mechanism of action is compulsory to bring compounds forward in the drug development pipeline. So here we focused on the identification of the nuclear and membrane targets of the two top ranking compounds Clobetasol and Halcinonide. Importantly, we were able to clarify that they act through the up-regulation of *RxRγ* gene expression. Indeed, the RxRγ inhibitor UVI3003 strongly reduces MBP expression in Clobetasol- and Halcinonide-treated *Oli-neuM* cells. Interestingly, we observed that the specific action of group I FGCs on MBP expression can be differentiated by the relative amount of *RxRγ* and *Gli1* gene expression promoted by each compound. Halcinonide, Amcinonide and Fluorandrenolide activate the transcription of both of them while Clobetasol and Fluticasone promote mainly *RxRγ* up-regulation. These observations add important information to the previous report regarding the action of Clobetasol [50] in that we show that Clobetasol requires *Smo* activation and *RxRγ* up-regulation. Surprisingly, Clobetasol and Halcinonide activation of *Smo*-mediated signaling does not increase the glucocorticoid receptor NR3C1 expression, although it has been reported that Clobetasol affects GR phosphorylation [50]. Putting together ours and the results of Najm et al. [50] it is tempting to speculate that Clobetasol acts at multiple levels on nuclear receptors. Compared to other studies analyzing the response to GCs at the genomic level, that highlighted the role of glucocorticoid receptor NR3C1 in response to GCs, it should be noted that they were mainly performed using Dexamethasone or other GCs of class II or III in our classification scheme [48, 51]. Thus the effect of GCs of class I on *RxRγ* expression might have been underscored, either due to the low activity of GCs of group II and III on RxRγ and/or by the fact that they used a different cellular model or treatment time points for analyses. The fact that efficient re-myelination in chronic MS patients depends largely on the ability of post-mitotic OPCs to up-regulate *RxRγ* at lesions, and OPC culture treatment with RxRγ antagonists leads to impaired OPC maturation [21], supports the view that these findings can be extended to premyelinating OLs.

Despite extensive analyses, it remains unclear which is the physiological activator of RxRγ at demyelinated regions [7, 21, 52]. Our data are consistent with the view that group I FGCs might activate *RxRγ* by interacting with *Smo* at cellular membrane. However, we cannot exclude that the two event are independent. Further experiments are required to clarify this point. However, it is clear that Clobetasol and Halcinonide activate MBP expression via Smoothened receptor activation. Cyclopamine and Itraconazole significantly inhibit MBP expression in Clobetasol- or Halcinonide-treated Oli-neuM cells. The observation that *Smo* activation is required for OL maturation at the site of remyelination in demyelinating cellular models [16, 42–45]. The morphogenetic Sonic Hedgehog (Shh) administrated exogenously increases the number of pro-myelinating OLs in the normal cerebellar cortex and corpus callosus in mice [53]. Moreover, *Smo* has a well-established role in regulating stem cell differentiation and stimulates primary neuronal precursor cell proliferation via canonical and non-canonical pathways [39, 53, 54]. Shh is required for establishment of the adult stem cell niche [55–57], the migration of neuroblasts [58], and Shh up-regulation is promoted by administration of interferon-beta [59]. Importantly, Shh up-regulation occurs at lesions in lysolecitin (LPC)-induced focal demyelination in wild type and *plp-GFP* transgenic mice. Shh up-regulation results in OPC proliferation, and Shh antagonists impair myelin repair [42]. It is also interesting to note that conditional activation of the Shh signaling pathway leads to an increase in adult neuronal stem cells in a NOTCH-mediated pathway [43]. The authors suggest that there is cross-talk between NOTCH and Hedgehog signaling, both contributing to properly maintain adult neuronal stem cell pools.

Regarding the mechanism of how Halcinonide, Clobetasol and possibly Fluticasone could modulate myelin gene expression by activating *Smo*, we postulated, based on current literature, that two pathways could be involved (Fig 5C): 1) direct up-regulation of RxRγ gene expression via *Smo* activation and 2) up-regulation of MBP mRNA translation at endosomes via the ability of *Smo* to activate the *Src*-related kinase Fyn [32, 40]. These two pathways are not mutually exclusive. Regarding the possibility that *Src* activation might also be part of the signaling pathway activated by FGCs acting on MBP expression, our preliminary study would exclude this hypothesis. The pan-Src kinase inhibitor PP2 does not affect MBP protein expression in Dexamethasone, Clobetasol or Halcinonide treated cells (S4 Fig) while Prednisone-treated cells respond significantly to PP2-treatment. *Smo*-mediated RxRγ up-regulation might be then modulated at lesions by other input signals. It has been suggested that the ability of the RxRγ to heterodimerize with a number of other nuclear receptors, responding to steroid or vitamin D might modulate gene expression during OL maturation [52]. This is an interesting idea and it might explain the different observations, as it remains unclear which nuclear receptor(s) might heterodimerize with RxRγ in OL lineage cells during remyelination processes [21]. A possible correlation between Vitamin D metabolism defects and MS and between Vitamin D3 and remyelination have been envisaged [60, 61] through Vitamin D Receptor (VDR) dimerization with RxRγ [6]. In addition to VDRs, RxRγ can potentially form heterodimers with receptors involved in cholesterol homeostasis and drug metabolism [62,63]. Najm et al. [50] reported that GRs are phosphorylated in the presence of Clobetasol and treatment with RU486 blocks OPC differentiation and GR phosphorylation 72h after treatment. Cross-talk between RxRγ and GRs at promoters of CYPs has been reported [49]. Thus it cannot be excluded that all transcription factors described so far converge on MBP expression in premyelinating OLs via the formation of heterodimers with RxRγ. Our study suggest that, if this is the case, the ability of a compound to up-regulate only RxRγ or also promote other nuclear receptor up-regulation might determine their final efficacy in myelin gene expression.

From the clinical point of view the reclassification of GCs performed in this study according to their pro-myelination properties might help in their choice for the treatment of neurological pathologies and the use of alternative compounds in the event of patient resistance or susceptibility. Regarding safety issues for GCs used in the clinic, although FGCs acting as *Smo* agonists are suggested for the treatment of neovascularization after myocardial infarction [64], neuronal degeneration after spinal cord injury [65], and wound healing in diabetes [66], we are not aware of data regarding their use for demyelinating diseases in the clinic. It should be mentioned also that synthetic *Smo* agonists are under observation for safety issues, since activating mutations of *Smo* are associated with the development of multiple type of cancers [67]. However, the safety profile of Clobetasol and Halcinonide has been known for decades, so such an issue cannot be applied to these specific *Smo* agonists [68]. Dexamethasone is one of the most widely used corticosteroids for the management of acute MS exacerbations due to its ability to reduce CNS inflammation and close the blood-brain barrier. However, it shows adverse effects upon administration in preterm and postnatal infants by increasing the risk of brain lesions or neuromotor defects [69–72]. Methylprednisolone is widely used as an anti-inflammatory drug to treat a wide range of human pathological conditions of the CNS consequent to autoimmune attacks, including MS [4, 72]. How Dexamethasone or Methylprednisolone function in OPC-mediated myelination remains controversial. Moreover, the use of Hydrocortisone in place of Dexamethasone in infants has been suggested because of its reduced neurotoxicity [73–75]. Our data suggest that Clobetasol and Halcinonide might be considered as an alternative to Dexamethasone or Methylprednisolone in MS therapy due to their safety and regenerative properties on myelin. Within the context of a safer use of GCs in neurodegenerative diseases, the reclassification of GCs according to their pro-myelination properties might help clinicians. Further studies in cellular and animal models are clearly needed to clarify how the top ranking GCs act on CNS remyelination.

In conclusion, our study identifies the targets at the plasma membrane of Clobetasol when acts in promoting MBP expression, namely Smoothened and we identified an effect of this compound on *RxRγ* gene transcription. Further work will clarify if these pathways leading to myelination are interconnected. Last but not least, our work provides novel indications for GC compounds use in the CNS regenerative therapy of MS and a novel cell-based assay to be used for screenings compounds acting in myelination.

## Supporting Information

S1 Fig Glucocorticoids stimulate MBP protein expression in *Oli-neuM* cells. (PDF)

S2 Fig Effect of glucocorticoids on PLP (DM20) and CNPase protein expression in *Oli-neuM* cells. (PDF)

S3 Fig Network analysis of hit compound protein targets. (PDF)

S4 Fig Fyn inhibitor PP2 does not influence MBP protein expression. (PDF)

S1 Table Glucocorticoid screening assay: quality and reproducibility statistical analyses. (DOCX)

S2 Table Glucocorticoid dose-response titration: MBP levels and arborization parameters. (PDF)

S3 Table Predicted functional partners of drug hits identified in primary screening. (DOCX)
