# Supplementary material for: Clobetasol and Halcinonide Act as Smoothened Agonists to Promote Myelin Gene Expression and RxRγ Receptor Activation
Source: PLoS One. 2015 Dec 10;10(12):e0144550. doi: 10.1371/journal.pone.0144550 (PMC4689554; doi:10.1371/journal.pone.0144550)
Supplement: S1 Fig — (PDF) [file pone.0144550.s001.pdf]

S1 Figure. Glucocorticoids stimulate MBP protein expression in Oli-neuM cells

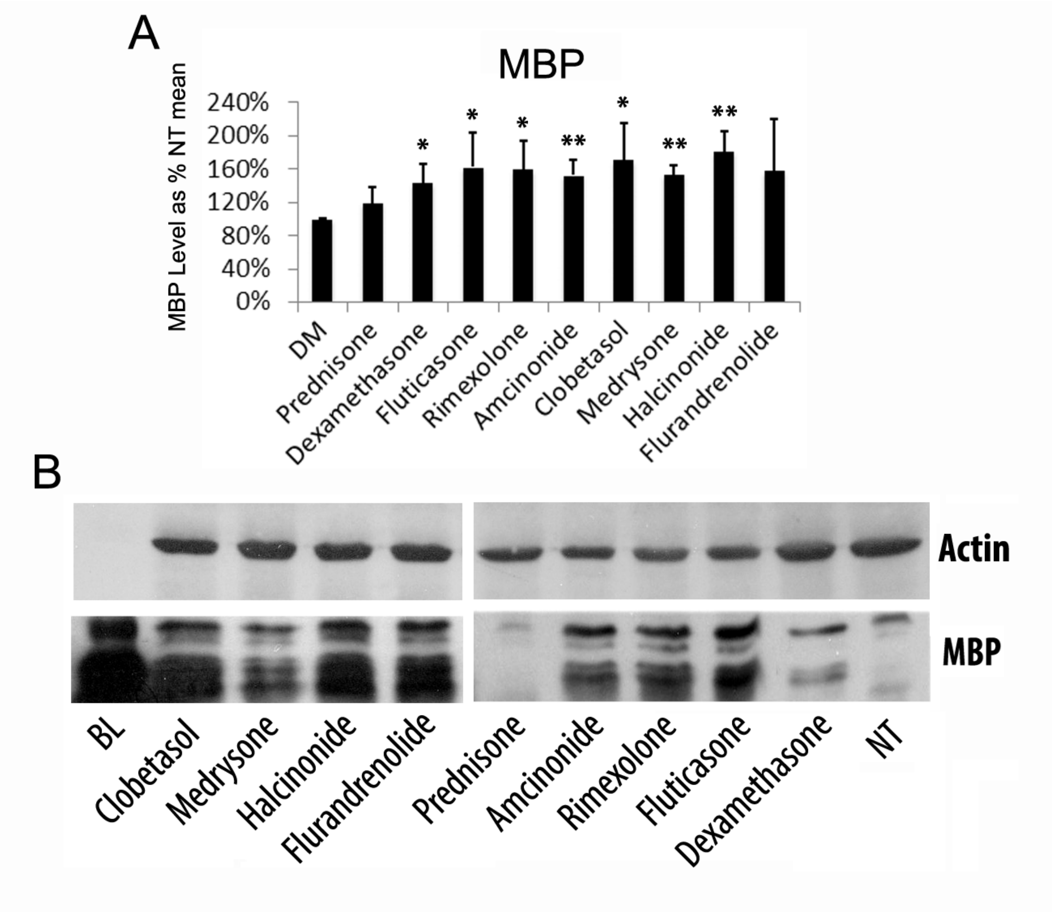

S1 Figure legend. Glucocorticoids stimulate MBP protein expression in Oli-neuM cells. (A) Graph indicating the relative MBP band intensities normalized with that of actin and expressed as % of variation compared to NT Oli-neuM cells were treated with 10 µM GCs for 48h. Data are presented as the mean ± SD (n = 5) and statistical significance was analysed by a two-tailed Student's t test with: Dexamethasone P = 0.0257, Fluticasone P = 0.05, Rimexolone P = 0.0408, Amcinonide P = 0.007, Clobetasol P = 0.05, Medrysone P = 0.001 and Halcinonide P = 0.004. (B) Representative immunoblot of the data in (A) (BL = Brain Lysate).*p≤0.05; **p≤0.01 No asterisk means p>0.05.
