# Supplementary material for: Clobetasol and Halcinonide Act as Smoothened Agonists to Promote Myelin Gene Expression and RxRγ Receptor Activation
Source: PLoS One. 2015 Dec 10;10(12):e0144550. doi: 10.1371/journal.pone.0144550 (PMC4689554; doi:10.1371/journal.pone.0144550)
Supplement: S2 Fig — (PDF) [file pone.0144550.s002.pdf]

S2 Figure. Effect of glucocorticoids on PLP (DM20) and CNPase protein expression in Oli-neuM cells

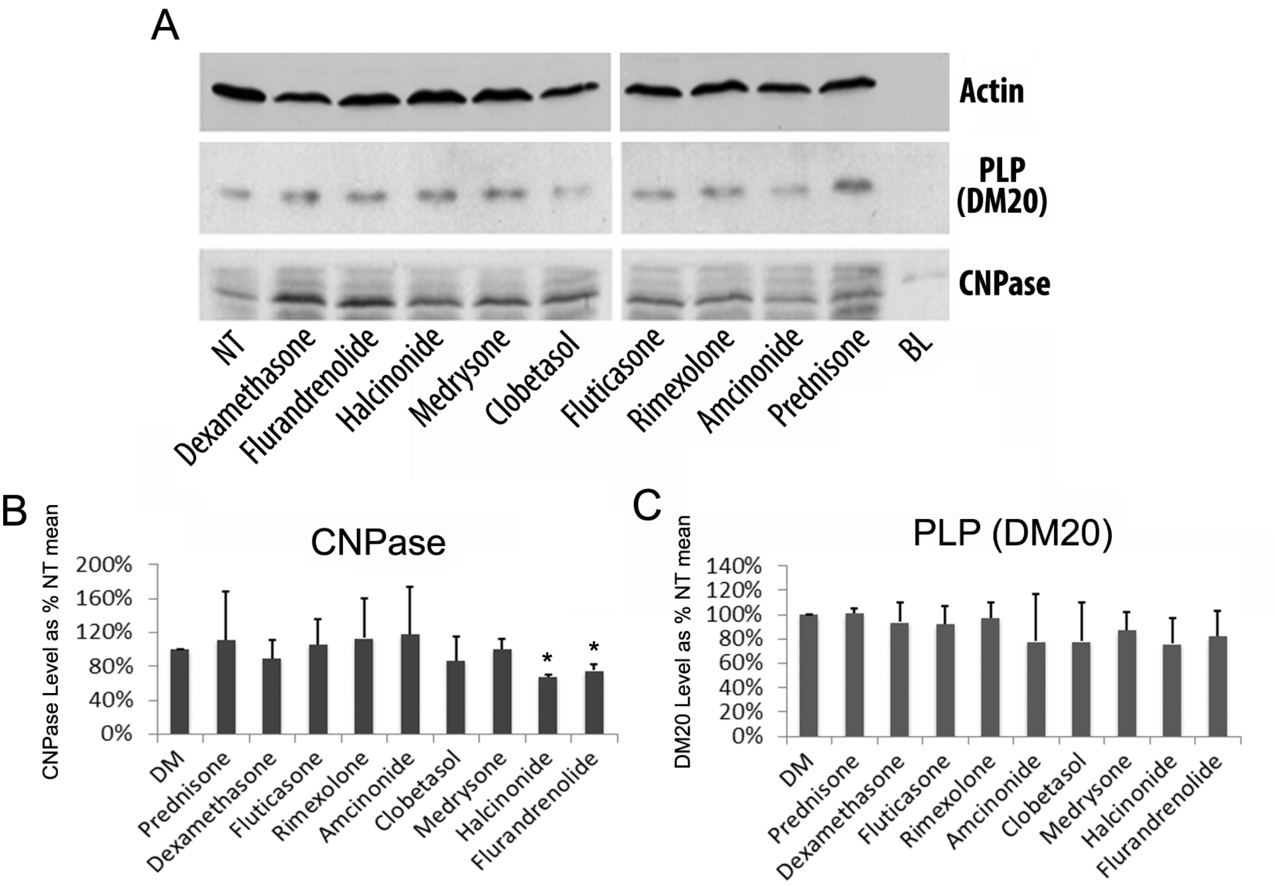

S2 Figure legend. Effect of glucocorticoids on PLP (DM20) and CNPase protein expression in Oli-neuM cells. (A) Representative immunoblot of PLP (DM20) and CNPase expressed in Oli-neuM cells treated with 10 µM GCs for 48h. Graphs indicate the relative CNPase (B) and PLP (DM20) (C) band intensities normalized with that of Actin and expressed as % of variation compared to NT. Data are presented as the mean ± SD (n = 5) and statistical significance was analysed by a two-tailed Student's t test with the following values for CNPase: Halcinonide $P < 0.0001$ and Flurandrenolide $P = 0.003$ (BL = Brain Lysate).
