# Supplementary material for: Clobetasol and Halcinonide Act as Smoothened Agonists to Promote Myelin Gene Expression and RxRγ Receptor Activation
Source: PLoS One. 2015 Dec 10;10(12):e0144550. doi: 10.1371/journal.pone.0144550 (PMC4689554; doi:10.1371/journal.pone.0144550)
Supplement: S3 Fig — (PDF) [file pone.0144550.s003.pdf]

S3 Figure. Network analysis of Hit compounds protein targets

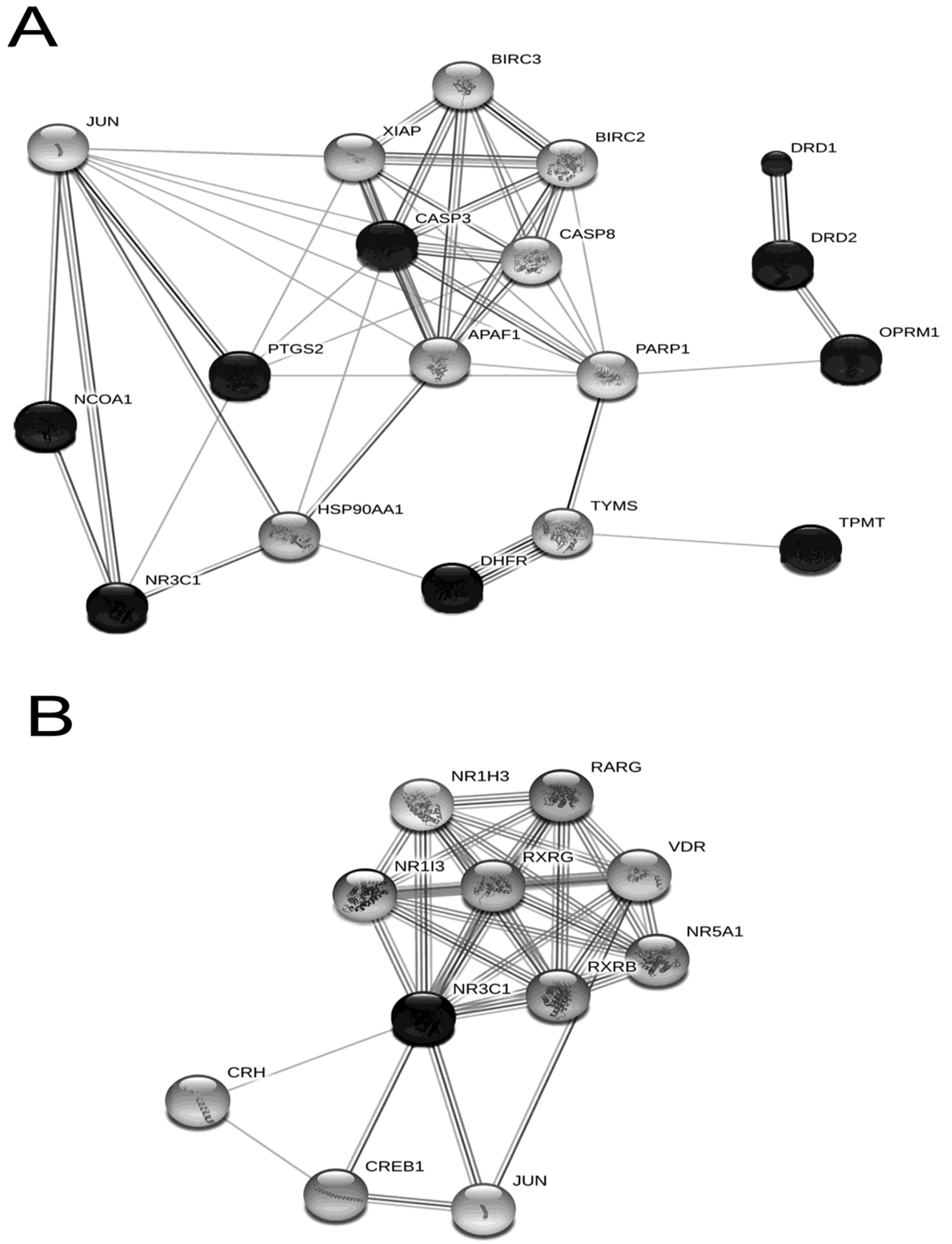

S3 Figure. **Hit compounds protein targets network analysis**. (A) Protein network derived from the analysis of protein targets of hit compounds. (B) Analysis of protein network formed by NR3C1 and the retinoid X receptor RxRγ. Network analysis were performed using String software (http://string.embl.de/). Drug hit target genes are highlighted in black, stronger associations are represented by thicker lines.
