# Supplementary material for: Clobetasol and Halcinonide Act as Smoothened Agonists to Promote Myelin Gene Expression and RxRγ Receptor Activation
Source: PLoS One. 2015 Dec 10;10(12):e0144550. doi: 10.1371/journal.pone.0144550 (PMC4689554; doi:10.1371/journal.pone.0144550)
Supplement: S4 Fig — (PDF) [file pone.0144550.s004.pdf]

S4 Figure. Fyn inhibitor PP2 does not influence MBP protein expression

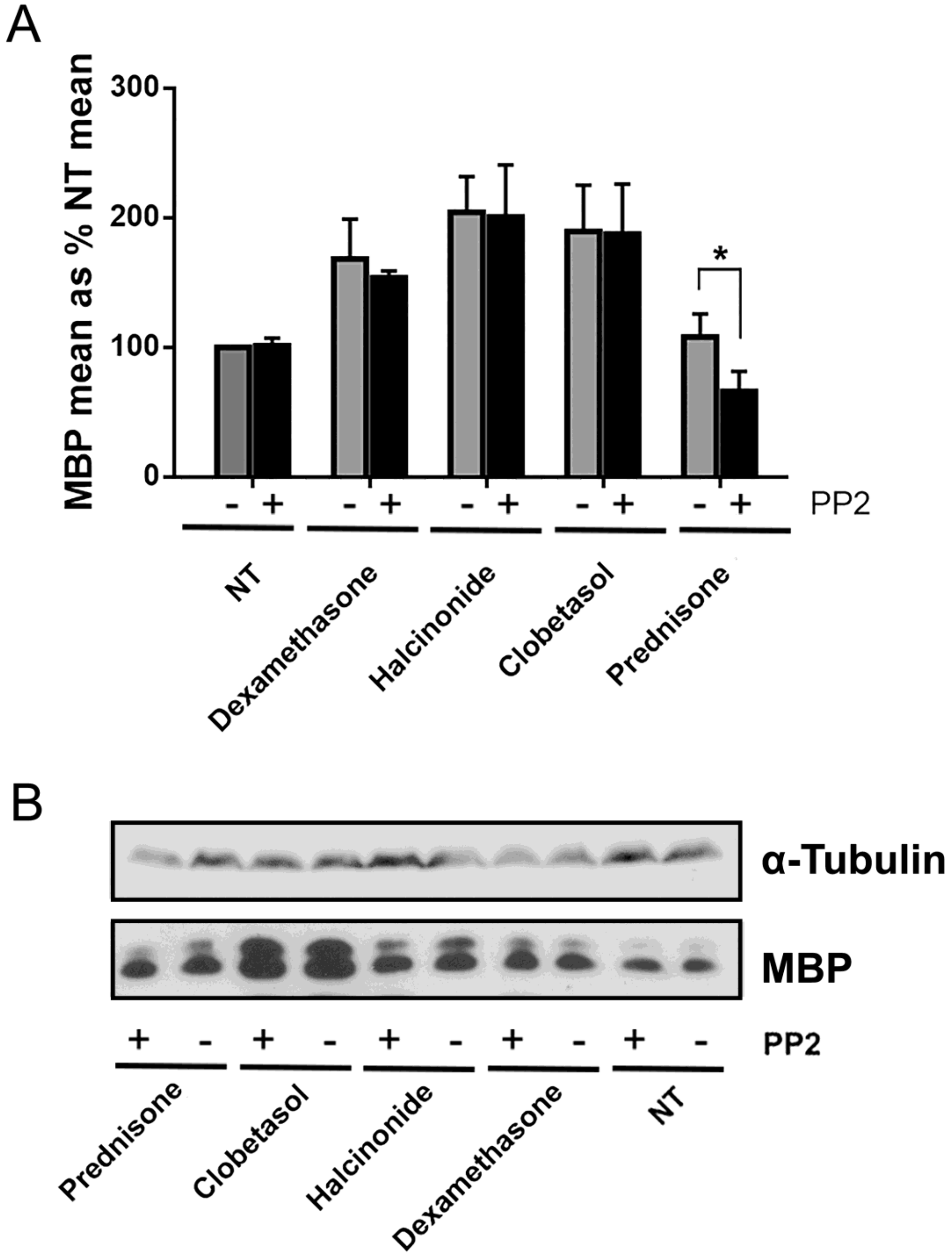

S4 Figure Legend. Fyn inhibitor PP2 does not influence MBP protein expression. (A) Graph indicates the MBP band intensities normalized with the α-Tubulin and express as % of variation compared to NT. (B) Representative immunoblot of Oli-neuM cells treated with 10 µM Glucocorticoids and 10µM PP2 for 48h. Data are presented as the mean ± SD (n = 3) and statistical significance was analysed by a Student's t test with *P = 0.03262.
