# Supplementary material for: Clobetasol and Halcinonide Act as Smoothened Agonists to Promote Myelin Gene Expression and RxRγ Receptor Activation
Source: PLoS One. 2015 Dec 10;10(12):e0144550. doi: 10.1371/journal.pone.0144550 (PMC4689554; doi:10.1371/journal.pone.0144550)
Supplement: S1 Table — (DOCX) [file pone.0144550.s005.docx]

**Table S1**. Glucocorticoids screening assay quality and reproducibility statistical analyses.

| **Compound** | **First Screening** | | | | **Second Screening** | | | | **Compound** | **Mean screenings as % of DM mean** | **Std Dev** | **Ponderate mean screenings of DM mean** |  | | |
| --- | --- | --- | --- | --- | --- | --- | --- | --- | --- | --- | --- | --- | --- | --- | --- |
|  | **Mean FITC as % of DM mean** | **CV% as Std Dev on DM mean** | **n. cells** | **Pr > t** | **Mean FITC as % of DM mean** | **CV% as Std Dev on DM mean** | **n. cells** | **Pr > t** |  |  |  |  | **Pooled Std Dev of DM mean** | **n.cells tot** | **Pr > t** |
|  |  |  |  |  |  |  |  |  | DM | 100 | 0 |  |  |  |  |
|  |  |  |  |  |  |  |  |  | Dexa 1uM | 150.3 | 14.76 | 153.66 | 112.72 | 10284 | 0.00E+00 |
|  |  |  |  |  |  |  |  |  | Dexa 10uM | 134.95 | 15.22 | 136.65 | 100.54 | 16179 | 0.00E+00 |
|  |  |  |  |  |  |  |  |  | Dexa 25 uM | 131.43 | 16.45 | 132.41 | 94.72 | 9315 | 1.55E-226 |
| **01A07_1uM** | 155.21 | 98.39 | 34 | 1.25E-03 | 157.21 | 123.22 | 1218 | 6.34E-54 | 01A07_1uM | 156.21 | 1.42 | 157.16 | 122.61 | 1252 | 9.27E-56 |
| **01A07_10uM** | 220.04 | 198.02 | 22 | 4.87E-03 | 142.98 | 109.37 | 2772 | 7.74E-89 | 01A07_10uM | 181.51 | 54.5 | 143.58 | 110.34 | 2794 | 2.30E-90 |
| **01A07_25uM** | 306 | 312.11 | 18 | 6.15E-03 | 140.1 | 107.89 | 2581 | 6.75E-75 | 01A07_25uM | 223.05 | 117.31 | 141.25 | 110.61 | 2599 | 7.22E-76 |
| **01H08_1uM** | 69.15 | 37.6 | 39 | 1.00E+00 | 119.54 | 99.88 | 703 | 1.39E-07 | 01H08_1uM | 94.35 | 35.63 | 116.89 | 97.6 | 742 | 1.44E-06 |
| **01H08_10uM** | 115.26 | 79.65 | 57 | 7.68E-02 | 102.5 | 83.06 | 725 | 2.09E-01 | 01H08_10uM | 108.88 | 9.02 | 103.43 | 82.82 | 782 | 1.24E-01 |
| **01H08_25uM** | 77.64 | 54.5 | 27 | 1.00E+00 | 108.64 | 86.27 | 1451 | 7.10E-05 | 01H08_25uM | 93.14 | 21.92 | 108.07 | 85.79 | 1478 | 1.54E-04 |
| **02D11_1uM** | 278.04 | 311.6 | 421 | 5.91E-28 | 157.64 | 110.56 | 2815 | 1.78E-149 | 02D11_1uM | 217.84 | 85.13 | 173.3 | 152.53 | 3236 | 1.59E-148 |
| **02D11_10uM** | 207.55 | 197.47 | 409 | 3.52E-25 | 133.42 | 102.48 | 3008 | 2.19E-68 | 02D11_10uM | 170.49 | 52.42 | 142.3 | 117.95 | 3417 | 3.52E-92 |
| **02D11_25uM** | 223.98 | 267.07 | 508 | 1.25E-23 | 140.99 | 103.24 | 2634 | 3.85E-86 | 02D11_25uM | 182.49 | 58.68 | 154.41 | 143.06 | 3142 | 1.35E-94 |
| **02E11_1uM** | 238.27 | 303.63 | 428 | 1.42E-19 | 140.95 | 100.48 | 1541 | 9.98E-54 | 02E11_1uM | 189.61 | 68.81 | 162.11 | 167.16 | 1969 | 1.31E-57 |
| **02E11_10uM** | 210.52 | 210.09 | 307 | 2.54E-18 | 141.72 | 109.02 | 2564 | 1.84E-78 | 02E11_10uM | 176.12 | 48.65 | 149.07 | 123.83 | 2871 | 2.32E-93 |
| **02E11_25uM** | 233.53 | 290.92 | 353 | 1.12E-16 | 128.34 | 98.93 | 2940 | 1.17E-52 | 02E11_25uM | 180.94 | 74.38 | 139.62 | 133.45 | 3293 | 9.89E-63 |
| **04E02_1uM** | 239.72 | 367.19 | 706 | 7.81E-23 | 141.93 | 106.72 | 1808 | 1.13E-58 | 04E02_1uM | 190.82 | 69.15 | 169.39 | 214.6 | 2514 | 1.33E-56 |
| **04E02_10uM** | 202.37 | 202.4 | 1142 | 8.58E-59 | 92.5 | 69.76 | 567 | 1.00E+00 | 04E02_10uM | 147.44 | 77.69 | 165.92 | 170.26 | 1709 | 3.92E-54 |
| **04E02_25uM** | 197.47 | 217.09 | 2200 | 3.89E-90 | 143.89 | 96.63 | 2154 | 4.12E-90 | 04E02_25uM | 170.68 | 37.89 | 170.96 | 168.62 | 4354 | 1.09E-156 |
| **04D05_1uM** | 257.39 | 291.76 | 1363 | 5.86E-78 | 129.3 | 93.83 | 2034 | 2.35E-43 | 04D05_1uM | 193.34 | 90.57 | 180.69 | 198.56 | 3397 | 3.22E-115 |
| **04D05_10uM** | 210.59 | 291.56 | 2583 | 8.82E-78 | 131.54 | 100.39 | 3086 | 1.93E-65 | 04D05_10uM | 171.07 | 55.9 | 167.56 | 210.28 | 5669 | 2.00E-123 |
| **04D05_25uM** | 205.91 | 299.72 | 2618 | 2.98E-69 | 145.33 | 101.83 | 2481 | 8.08E-100 | 04D05_25uM | 175.62 | 42.84 | 176.43 | 226.2 | 5099 | 3.41E-122 |
| **04D10_1uM** | 244.31 | 366 | 1602 | 1.47E-52 | 135.52 | 100.23 | 2210 | 4.30E-59 | 04D10_1uM | 189.91 | 76.92 | 181.24 | 249.24 | 3812 | 5.51E-86 |
| **04D10_10uM** | 225.69 | 251.41 | 1132 | 3.83E-57 | 129 | 95.09 | 2787 | 3.87E-56 | 04D10_10uM | 177.35 | 68.37 | 156.93 | 157.12 | 3919 | 1.99E-107 |
| **04D10_25uM** | 244.68 | 369.23 | 535 | 1.19E-18 | 127.12 | 103.89 | 2855 | 4.07E-43 | 04D10_25uM | 185.9 | 83.13 | 145.67 | 174.94 | 3390 | 7.90E-51 |
| **05E03_1uM** | 198.03 | 197.77 | 691 | 3.71E-35 | 144.68 | 104.92 | 1806 | 1.07E-67 | 05E03_1uM | 171.36 | 37.73 | 159.44 | 137.06 | 2497 | 7.13E-96 |
| **05E03_10uM** | 190.02 | 280.75 | 807 | 3.23E-19 | 131.3 | 102.81 | 1911 | 5.27E-39 | 05E03_10uM | 160.66 | 41.52 | 148.73 | 175.6 | 2718 | 4.61E-46 |
| **05E03_25uM** | 189.87 | 243.4 | 544 | 3.92E-17 | 138.29 | 98.38 | 1706 | 1.51E-54 | 05E03_25uM | 164.08 | 36.47 | 150.76 | 147.18 | 2250 | 3.20E-57 |
| **06D03_1uM** | 127.31 | 113.89 | 1027 | 1.80E-14 | 142 | 104.64 | 2508 | 9.14E-84 | 06D03_1uM | 128.66 | 12.72 | 137.73 | 107.41 | 3535 | 9.28E-92 |
| **06D03_10uM** | 140.87 | 131.71 | 2896 | 3.73E-60 | 142.17 | 114.97 | 2404 | 3.04E-68 | 06D03_10uM | 141.69 | 0.71 | 141.46 | 124.4 | 5300 | 9.94E-124 |
| **06D03_25uM** | 135.03 | 129.75 | 1495 | 5.75E-25 | 143.29 | 101.22 | 2491 | 2.76E-93 | 06D03_25uM | 134.28 | 9.42 | 140.19 | 112.77 | 3986 | 5.94E-106 |
| **06E08_1uM** | 148.48 | 149.7 | 4056 | 2.91E-90 | 142.41 | 103.52 | 2225 | 2.50E-77 | 06E08_1uM | 137.59 | 13.94 | 146.33 | 135.16 | 6281 | 4.72E-154 |
| **06E08_10uM** | 131.58 | 117.29 | 3543 | 3.67E-56 | 133.3 | 101.93 | 2377 | 1.17E-54 | 06E08_10uM | 122.85 | 16.63 | 132.27 | 111.37 | 5920 | 4.30E-106 |
| **06E08_25uM** | 145.29 | 144.6 | 3191 | 3.34E-67 | 135.14 | 99.02 | 2382 | 1.12E-63 | 06E08_25uM | 133.68 | 12.41 | 140.95 | 127.14 | 5573 | 6.00E-122 |
| **07D09_1uM** | 199.46 | 225.13 | 1398 | 2.00E-56 | 150.93 | 108.52 | 1972 | 1.18E-87 | 07D09_1uM | 175.2 | 34.31 | 171.06 | 167.08 | 3370 | 3.72E-124 |
| **07D09_10uM** | 183.65 | 180.82 | 3010 | 3.09E-129 | 136.22 | 95.07 | 2138 | 2.84E-65 | 07D09_10uM | 159.94 | 33.54 | 163.95 | 151.23 | 5148 | 1.76E-186 |
| **07D09_25uM** | 297.34 | 356.53 | 285 | 1.42E-18 | 141.02 | 109.94 | 316 | 7.19E-11 | 07D09_25uM | 219.18 | 110.54 | 215.15 | 258.14 | 601 | 8.27E-26 |
| **09A04_1uM** | 170.84 | 138.52 | 992 | 2.54E-52 | 159.74 | 115.55 | 2070 | 6.48E-109 | 09A04_1uM | 165.29 | 7.85 | 163.33 | 123.46 | 3062 | 7.43E-158 |
| **09A04_10uM** | 157.14 | 139.69 | 2606 | 5.78E-90 | 128.16 | 96.05 | 3114 | 4.28E-58 | 09A04_10uM | 142.65 | 20.49 | 141.37 | 117.95 | 5720 | 1.34E-146 |
| **09A04_25uM** | 201.58 | 200.53 | 2149 | 5.05E-109 | 146.29 | 110.57 | 3063 | 7.41E-110 | 09A04_25uM | 173.93 | 39.1 | 169.09 | 154.16 | 5212 | 1.02E-209 |
| **09A06_1uM** | 170.19 | 146.46 | 1212 | 1.02E-56 | 165.09 | 122.04 | 1818 | 2.97E-101 | 09A06_1uM | 167.64 | 3.61 | 167.13 | 132.35 | 3030 | 3.70E-153 |
| **09A06_10uM** | 158.59 | 133.59 | 1041 | 5.32E-42 | 152.5 | 112.72 | 2640 | 5.03E-115 | 09A06_10uM | 155.54 | 4.3 | 154.22 | 118.99 | 3681 | 2.45E-153 |
| **09A06_25uM** | 154.34 | 133.43 | 991 | 3.23E-35 | 143.88 | 103.6 | 2791 | 2.03E-102 | 09A06_25uM | 149.11 | 7.4 | 146.62 | 112.19 | 3782 | 2.65E-133 |
| **09B06_1uM** | 162.84 | 144.3 | 947 | 6.31E-38 | 148.98 | 102.48 | 1805 | 5.21E-83 | 09B06_1uM | 155.91 | 9.8 | 153.75 | 118.55 | 2752 | 3.60E-114 |
| **09B06_10uM** | 189.33 | 154.43 | 856 | 6.17E-56 | 162.84 | 115.37 | 1314 | 1.71E-76 | 09B06_10uM | 176.09 | 18.73 | 173.29 | 132.16 | 2170 | 8.09E-129 |
| **09B06_25uM** | 169.39 | 143.72 | 381 | 2.18E-19 | 161.32 | 111.71 | 914 | 1.48E-54 | 09B06_25uM | 165.35 | 5.7 | 163.69 | 122 | 1295 | 4.07E-70 |
| **09H09_1uM** | 132.34 | 106.92 | 1746 | 2.10E-35 | 131.61 | 96.32 | 2308 | 1.47E-53 | 09H09_1uM | 131.55 | 0.83 | 131.92 | 101.02 | 4054 | 3.35E-86 |
| **09H09_10uM** | 99.99 | 93.55 | 1499 | 1.00E+00 | 127.21 | 99.4 | 2904 | 7.64E-48 | 09H09_10uM | 116.12 | 14.29 | 117.94 | 97.45 | 4403 | 4.38E-34 |
| **09H09_25uM** | 120.94 | 114.36 | 1863 | 2.29E-15 | 133.31 | 96.36 | 2296 | 1.31E-58 | 09H09_25uM | 128.22 | 6.47 | 127.77 | 104.81 | 4159 | 1.25E-63 |
| **10C04_1uM** | 139.14 | 116.27 | 608 | 3.36E-16 | 152.88 | 112.6 | 1135 | 2.11E-51 | 10C04_1uM | 146.01 | 9.72 | 148.09 | 113.9 | 1743 | 1.99E-64 |
| **10C04_10uM** | 214.68 | 169.19 | 384 | 1.11E-33 | 154.07 | 109.14 | 1097 | 1.44E-54 | 10C04_10uM | 184.37 | 42.86 | 169.78 | 127.46 | 1481 | 1.06E-86 |
| **10C04_25uM** | 238.61 | 190.85 | 273 | 3.27E-27 | 178.17 | 116.47 | 436 | 2.20E-37 | 10C04_25uM | 208.39 | 42.74 | 201.44 | 149.56 | 709 | 1.48E-60 |
| **10G02_1uM** | 183.66 | 159.38 | 1625 | 2.84E-88 | 149.84 | 92.41 | 1195 | 1.37E-68 | 10G02_1uM | 166.75 | 23.92 | 169.33 | 135.11 | 2820 | 1.26E-145 |
| **10G02_10uM** | 280.82 | 185.71 | 1174 | 1.85E-172 | 164.9 | 102.35 | 904 | 1.17E-68 | 10G02_10uM | 222.86 | 81.97 | 230.39 | 155.05 | 2078 | 6.69E-244 |
| **10G02_25uM** | 288.05 | 188.31 | 933 | 1.43E-142 | 183.51 | 106.99 | 529 | 6.56E-57 | 10G02_25uM | 235.78 | 73.92 | 250.23 | 163.62 | 1462 | 1.34E-196 |
| **10B05_1uM** | 180.93 | 127.18 | 1254 | 5.52E-95 | 133.11 | 98.96 | 1819 | 3.59E-44 | 10B05_1uM | 157.02 | 33.81 | 152.63 | 111.34 | 3073 | 4.81E-137 |
| **10B05_10uM** | 157.7 | 109.51 | 1814 | 6.31E-99 | 127.79 | 98.39 | 2799 | 6.20E-49 | 10B05_10uM | 142.74 | 21.15 | 139.55 | 102.9 | 4613 | 1.58E-140 |
| **10B05_25uM** | 151.95 | 121.32 | 1338 | 3.36E-51 | 137.87 | 96.42 | 2721 | 3.68E-87 | 10B05_25uM | 144.91 | 9.96 | 142.51 | 105.28 | 4059 | 1.27E-135 |
| **10F05_1uM** | 168.64 | 134.73 | 1205 | 1.07E-62 | 131.52 | 97.88 | 1287 | 9.48E-30 | 10F05_1uM | 150.08 | 26.24 | 149.47 | 117.16 | 2492 | 3.38E-91 |
| **10F05_10uM** | 171.81 | 142.11 | 1372 | 4.23E-70 | 150.4 | 111.02 | 1659 | 7.44E-70 | 10F05_10uM | 161.11 | 15.14 | 160.09 | 126.04 | 3031 | 2.69E-137 |
| **10F05_25uM** | 174.98 | 148.23 | 1328 | 4.86E-68 | 124.07 | 89.95 | 1274 | 3.15E-21 | 10F05_25uM | 149.53 | 36 | 150.05 | 123.19 | 2602 | 9.59E-89 |
| **11B08_1uM** | 165.14 | 162.26 | 3184 | 9.22E-106 | 143.49 | 121.52 | 1383 | 2.07E-38 | 11B08_1uM | 141.15 | 25.24 | 158.58 | 151.09 | 4567 | 2.05E-141 |
| **11B08_10uM** | 152.28 | 146.53 | 3368 | 4.85E-90 | 127.07 | 93.44 | 1384 | 2.25E-26 | 11B08_10uM | 129.35 | 21.88 | 144.94 | 133.27 | 4752 | 1.37E-113 |
| **11B08_25uM** | 158.34 | 192.33 | 3175 | 5.03E-63 | 130.86 | 98.31 | 1261 | 6.95E-28 | 11B08_25uM | 134.35 | 22.44 | 150.53 | 170.95 | 4436 | 4.41E-83 |
| **12B02_1uM** | 224.62 | 212.33 | 2369 | 9.02E-155 | 132.92 | 98.82 | 2368 | 1.99E-56 | 12B02_1uM | 178.77 | 64.84 | 178.78 | 165.61 | 4737 | 2.11E-212 |
| **12B02_10uM** | 178.52 | 183.72 | 3381 | 1.19E-125 | 150.6 | 115.77 | 1476 | 2.50E-58 | 12B02_10uM | 164.56 | 19.74 | 170.03 | 166.04 | 4857 | 3.01E-175 |
| **12B02_25uM** | 191.68 | 238.83 | 3377 | 3.06E-103 | 145.33 | 112.46 | 2131 | 4.80E-72 | 12B02_25uM | 168.51 | 32.77 | 173.75 | 199.66 | 5508 | 1.67E-155 |
| **13D08_1uM** | 168.34 | 151.35 | 1057 | 7.63E-45 | 159.37 | 111.65 | 630 | 2.95E-36 | 13D08_1uM | 163.86 | 6.35 | 164.99 | 137.87 | 1687 | 7.06E-76 |
| **13D08_10uM** | 170.05 | 149.92 | 1079 | 1.58E-48 | 148.31 | 113.64 | 1976 | 1.22E-73 | 13D08_10uM | 159.18 | 15.37 | 155.98 | 127.64 | 3055 | 3.38E-119 |
| **13D08_25uM** | 179.88 | 160.67 | 697 | 1.25E-35 | 158.93 | 118.14 | 435 | 4.40E-23 | 13D08_25uM | 169.41 | 14.82 | 171.83 | 145.8 | 1132 | 1.03E-55 |
| **13F11_1uM** | 197.05 | 209.18 | 68 | 1.44E-04 | 142.1 | 106 | 2237 | 1.64E-73 | 13F11_1uM | 169.58 | 38.86 | 143.72 | 110.43 | 2305 | 3.01E-75 |
| **13F11_10uM** | 183.09 | 180.66 | 205 | 1.88E-10 | 149.55 | 114.33 | 452 | 6.00E-19 | 13F11_10uM | 166.32 | 23.72 | 160.02 | 138.48 | 657 | 1.08E-26 |
| **13F11_25uM** | 197.78 | 177.95 | 547 | 1.64E-33 | 132.11 | 93.93 | 994 | 5.41E-26 | 13F11_25uM | 164.94 | 46.43 | 155.42 | 130.12 | 1541 | 4.30E-58 |
| **14A05_1uM** | 139.62 | 147.55 | 2140 | 1.48E-34 | 137.56 | 99.43 | 804 | 2.07E-25 | 14A05_1uM | 135.76 | 5 | 139.06 | 136.11 | 2944 | 6.97E-53 |
| **14A05_10uM** | 153.71 | 151.21 | 987 | 1.28E-27 | 139.12 | 100.27 | 1119 | 1.23E-36 | 14A05_10uM | 142.49 | 9.97 | 145.96 | 126.72 | 2106 | 7.84E-59 |
| **14A05_25uM** | 171.19 | 170.27 | 1093 | 1.80E-40 | 133.83 | 96.28 | 1757 | 1.12E-46 | 14A05_25uM | 143.43 | 24.42 | 148.16 | 129.74 | 2850 | 2.85E-82 |
| **14H10_1uM** | 179.41 | 160.27 | 1975 | 1.43E-96 | 100.79 | 71.95 | 2045 | 3.10E-01 | 14H10_1uM | 140.1 | 55.59 | 139.42 | 123.5 | 4020 | 4.40E-87 |
| **14H10_10uM** | 200.49 | 197.85 | 1030 | 1.29E-53 | 128.81 | 89.34 | 2071 | 9.40E-47 | 14H10_10uM | 164.65 | 50.69 | 152.62 | 135.4 | 3101 | 3.88E-97 |
| **14H10_25uM** | 190.6 | 192.04 | 1146 | 2.74E-52 | 114.45 | 80.47 | 1211 | 2.88E-10 | 14H10_25uM | 152.52 | 53.85 | 151.47 | 145.8 | 2357 | 1.89E-62 |
| **15B06_1uM** | 181.26 | 153.62 | 1232 | 2.41E-68 | 121.42 | 88.25 | 2409 | 3.76E-32 | 15B06_1uM | 151.34 | 42.31 | 141.67 | 114.62 | 3641 | 1.34E-100 |
| **15B06_10uM** | 223.03 | 205.01 | 206 | 9.47E-16 | 132.07 | 86.96 | 900 | 4.63E-27 | 15B06_10uM | 177.55 | 64.32 | 149.01 | 118.24 | 1106 | 2.64E-40 |
| **15B06_25uM** | 208.15 | 169.58 | 558 | 1.44E-43 | 144.36 | 91.08 | 1280 | 1.73E-61 | 15B06_25uM | 176.26 | 45.11 | 163.73 | 120.45 | 1838 | 6.32E-101 |
| **15E06_1uM** | 208.32 | 178.7 | 1320 | 4.29E-92 | 129.03 | 91.24 | 1229 | 6.96E-28 | 15E06_1uM | 168.68 | 56.07 | 170.09 | 143.36 | 2549 | 4.12E-121 |
| **15E06_10uM** | 185.73 | 151.69 | 1067 | 1.51E-66 | 137.2 | 96.11 | 1647 | 3.13E-52 | 15E06_10uM | 161.47 | 34.31 | 156.28 | 121.04 | 2714 | 8.16E-118 |
| **15E06_25uM** | 191.51 | 168.38 | 1282 | 2.13E-74 | 116.82 | 86.36 | 1589 | 7.30E-15 | 15E06_25uM | 154.17 | 52.81 | 150.17 | 129.57 | 2871 | 1.62E-89 |

1. Mean screening values were computed as weighted means of the first two screenings, using cell number as weights. For each compound a one sample two-sided t-test was performed. Columns headed Pr > t represent the P values of the correspondent two-tailed Student's t tests. Analyses were performed using SAS software (Release 9.3. Cary, NC, USA). (Compounds are expressed in Prestwick Chemical Library code; DM = Differentiation Medium, Dexa = Dexamethasone (02E11), 01A07 = Isoflupredone Acetate, 01H08 = Prednisone, 02D11 = Triamcinolone, 04E02 = Fludrocortisone Acetate, 04D05 = Prednisolone, 04D10 = Methylprednisolone 6-alpha, 05E03 = Bethametasone, 06D03 = Corticosterone, 06E08 = Hydrocortisone base, 07D09 = Budesonide, 09A04 = Flunisolide, 09A06 = Flurandrenolide, 09B06 = Halcinonide, 09H09 = Fluorometholone, 10C04 = Medrysone, 10G02 = Clobetasol Propionate, 10B05 = Flumethasone, 10F05 = Fluocinonide, 11B08 = Deflazacort, 12B02 = Cortisol Acetate, 13D08 = Fluticasone Propionate, 13F11 = Rimexolone, 14A05 = Prednicarbate, 14H10 = Clocortolone Pivalate, 15B06 = Amcinonide, 15E06 = Fluocinolone Acetonide).
