# Supplementary material for: Clobetasol and Halcinonide Act as Smoothened Agonists to Promote Myelin Gene Expression and RxRγ Receptor Activation
Source: PLoS One. 2015 Dec 10;10(12):e0144550. doi: 10.1371/journal.pone.0144550 (PMC4689554; doi:10.1371/journal.pone.0144550)
Supplement: S2 Table — (PDF) [file pone.0144550.s006.pdf]

Table S2. Glucocorticoid dose-response titration experiments: MBP (table S2A) and arborization (table S2B). parameters

Table S2A. Dose-response titration experiments based on MBP signal. MBP levels were calculated based on the MEAN intensity FITC signal in main by the Olympus Scan[R] Analysis Software. *Oli-neuM* cells treated with GCs at the indicated concentrations were plated in 96-well plates as indicated in the text. Values in bold represent the drug EC_{50} referring to the sigmoid dose-response represented in Fig 4B. Data represent the mean of two independent experiments performed in triplicate in each plate and data were normalized using NT control conditions for each plate.

Log concentration (μM)	Clobetasol	Dexamethasone	Prednisone	Fluticasone	Flurandrenolide	Halcinonide	Medrysone	Amcinonide
**-6**	188.7069	98.5112	154.1632	165.8993	108.33	164.4014	124.8448	174.5307
**-5**	191.2488	97.09866	130.7745	**204.096**	105.1091	179.5817	118.6953	172.8585
**-4**	207.72	111.4928	129.6234	189.5276	131.5952	180.3642	105.1963	173.8208
**-3**	200.5011	**111.5872**	**97.52351**	/	148.6002	184.2514	109.2632	123.011
**-2**	213.3883	200.5519	92.42165	/	120.6362	188.411	135.1338	**184.4893**
**-1**	**216.4494**	138.4727	99.77123	212.397	**191.2161**	**190.1049**	114.7082	167.3935
**-1.5**	/	195.5663	98.92153	/	185.9872	180.6947	143.5274	220.0734
**0**	343.5201	191.5429	90.90381	238.8939	248.1463	239.1989	**162.9616**	194.4679
**1**	307.9197	224.2892	/	198.5403	222.8004	207.8688	263.6352	157.68
**2**	/	200.8606	/	189.0011	/	173.8044	203.7329	/

Table S2B. Arborization parameters result table. Dose-response titration experiments based on actin and elongation parameters were performed using the indicated compounds at the following concentrations: 0.001 μM, 0.01 μM, 0.1 μM, 1 μM, 10 μM. The number 5 in the table indicates that at all five concentrations tested the indicated compound had a percentage (%) of arborized cells above that of Dexamethasone; 0 indicate a result always below Dexamethasone. A score from 1 to 4 indicates intermediate results. The color code was used in the table to easily visualize the data in Table S2. Glucocorticoid dose-response titration experiments for MBP (table S2A) and arborization parameters (table S2B).

Experimental details: Typically, *Oli-neuM* cells were plated for 48h prior to treatment in 96 well plates and samples were treated as indicated in the text in parallel experiments. The actin signal was acquired using an RFP filter and 20x Objective using the ScanR acquisition microscopy platform and using an anti-actin antibody/Alexa546-conjugated secondary antibody. After acquisition, mean intensity values of the actin signal and elongation parameters were plotted in a graph using the ScanR analysis software. Arborized cells were gated according to visual criteria and data were expressed as percentage of cells present in the arborization gate relative to DM. A score of 0 was given to any compound that had a percentage (%) of cells in gate arborization that was below that of dexamethasone at the same concentration and a score of 1 was given to those cells in the arborization gate above dexamethasone. Thus a score of 5 indicates that a given compound always had a % of cells above Dexamethasone at all concentrations tested while a score of 0 indicates that none were above Dexamethasone.

Arborization Test I	Test II	mean n=2	GC compounds
5	3	4	Halcinonide
3	3	3	Clobetasol
3	3	3	Fluoticasone
4	2	3	Fluticasone
2	1	1,5	Medrysone
1	1	1	Triamcinolone
1	0	0,5	Prednisone
