# Supplementary material for: Clobetasol and Halcinonide Act as Smoothened Agonists to Promote Myelin Gene Expression and RxRγ Receptor Activation
Source: PLoS One. 2015 Dec 10;10(12):e0144550. doi: 10.1371/journal.pone.0144550 (PMC4689554; doi:10.1371/journal.pone.0144550)
Supplement: S3 Table — (DOCX) [file pone.0144550.s007.docx]

**S3 Table.** Predicted functional partners of drug hits identified in primary screening.

| **Gene** | **Description** | **Function** | **Score** |
| --- | --- | --- | --- |
| Ptgs2 | Prostaglandin-endoperoxide synthase 2 gene prostaglandin G/H synthase and cyclooxygenase | May have a role as a major mediator of inflammation and/or a role for prostanoid signaling in activity-dependent plasticity | **0.999** |
| Nr3c1 | Nuclear receptor subfamily 3, group C, member 1 gene | Receptor for glucocorticoids (GC). Has a dual mode of action: as a transcription factor that binds to glucocorticoid response elements (GRE) and as a modulator of other transcription factors. Affects inflammatory responses, cellular proliferation and differentiation in target tissues. Could act as a coactivator for STAT5-dependent transcription upon growth hormone (GH) stimulation and could reveal an essential role of hepatic GR in the control of body growth. Involved in chromatin remodelling | **0.999** |
| Dhfr | Dihydrofolate reductase gene | Key enzyme in folate metabolism. Contributes to the de novo mitochondrial thymidylate biosynthesis pathway. Catalyzes an essential reaction for de novo glycine and purine synthesis, and for DNA precursor synthesis | **0.999** |
| Tpmt | Thiopurine methyltransferase | Catalyzes the S-methylation of thiopurine drugs such as 6-mercaptopurine and azathioprine | **0.998** |
| Htr2c | 5-hydroxytryptamine (serotonin) receptor 2C | This is one of the several different receptors for 5- hydroxytryptamine (serotonin), a biogenic hormone that functions as a neurotransmitter, a hormone, and a mitogen. This receptor mediates its action by association with G proteins that activate a phosphatidylinositol-calcium second messenger system | **0.996** |
| Oprm1: | Oppioid receptor, mu 1 Gene | Inhibits neurotransmitter release by reducing calcium ion currents and increasing potassium ion conductance. Receptor for beta-endorphin | **0.995** |
| Hmgcr3: | 3-hydroxy-3-methylglutaryl-Coenzyme A reductase | This transmembrane glycoprotein is involved in the control of cholesterol biosynthesis. It is the rate-limiting enzyme of sterol biosynthesis | **0.992** |
| Drd1 and Drd2 | Dopamine receptor gene 1 and 2 | This are two of the five types (D1 to D5) of receptors for dopamine. The activity of this receptor is mediated by G proteins which activate adenylyl cyclise | **0.998** |
| Casp3 | Caspase 3 | Involved in the activation cascade of caspases responsible for apoptosis execution. At the onset of apoptosis it proteolytically cleaves poly(ADP-ribose) polymerase (PARP) at a '216-Asp-\|-Gly-217' bond. Cleaves and activates sterol regulatory element binding proteins (Cleaves and activates caspase-6, -7 and -9) | **0.984** |
